# Biomarker Reproducibility Challenge: A Review of Non-Nucleotide Biomarker Discovery Protocols from Body Fluids in Breast Cancer Diagnosis

**DOI:** 10.3390/cancers15102780

**Published:** 2023-05-16

**Authors:** Fatemeh Safari, Cheka Kehelpannala, Azadeh Safarchi, Amani M. Batarseh, Fatemeh Vafaee

**Affiliations:** 1 School of Biotechnology and Biomolecular Sciences, University of New South Wales (UNSW Sydney), Sydney, NSW 2052, Australia; fatemeh.safari@unsw.edu.au (F.S.); a.safarchi@unsw.edu.au (A.S.); 2 BCAL Diagnostics Ltd., Suite 506, 50 Clarence St, Sydney, NSW 2000, Australia; clkehelpannala@gmail.com; 3 BCAL Dx, The University of Sydney, Sydney Knowledge Hub, Merewether Building, Sydney, NSW 2006, Australia; 4 Microbiomes for One Systems Health, Health and Biosecurity, CSIRO, Westmead, NSW 2145, Australia; 5 UNSW Data Science Hub (uDASH), University of New South Wales (UNSW Sydney), Sydney, NSW 2052, Australia; 6 OmniOmics.ai Pty Ltd., Sydney, NSW 2035, Australia

**Keywords:** reproducibility, proteomics, lipidomics, metabolomics, liquid biopsy, breast cancer

## Abstract

**Simple Summary:**

Various studies and techniques have been designed to discover biofluid-derived biomarkers for non-invasive early detection and prognosis of cancers. Despite the importance of non-invasive biomarker discovery in cancer diagnosis and management, the reported markers are often inconsistent and irreproducible across different studies and cohorts. In this article, we reviewed the ongoing trend of non-nucleotide biomarkers, including lipidomics, proteomics and metabolomics, derived from body fluids, with a focus on breast cancer, and reviewed the inconstancies in the biomarker discovery pipelines across pre-analytical, analytical, and post-analytical phases, covering the diversity of approaches from sample processing to predictive modelling and validation.

**Abstract:**

Breast cancer has now become the most commonly diagnosed cancer, accounting for one in eight cancer diagnoses worldwide. Non-invasive diagnostic biomarkers and associated tests are superlative candidates to complement or improve current approaches for screening, early diagnosis, or prognosis of breast cancer. Biomarkers detected from body fluids such as blood (serum/plasma), urine, saliva, nipple aspiration fluid, and tears can detect breast cancer at its early stages in a minimally invasive way. The advancements in high-throughput molecular profiling (omics) technologies have opened an unprecedented opportunity for unbiased biomarker detection. However, the irreproducibility of biomarkers and discrepancies of reported markers have remained a major roadblock to clinical implementation, demanding the investigation of contributing factors and the development of standardised biomarker discovery pipelines. A typical biomarker discovery workflow includes pre-analytical, analytical, and post-analytical phases, from sample collection to model development. Variations introduced during these steps impact the data quality and the reproducibility of the findings. Here, we present a comprehensive review of methodological variations in biomarker discovery studies in breast cancer, with a focus on non-nucleotide biomarkers (i.e., proteins, lipids, and metabolites), highlighting the pre-analytical to post-analytical variables, which may affect the accurate identification of biomarkers from body fluids.

## 1. Background

The number of women diagnosed with breast cancer is increasing every year [1]. Female breast cancer surpassed lung cancer as the most diagnosed cancer in the world in 2020, with approximately 2.3 million new cases diagnosed [2]. Although statistics show that the mortality rate of breast cancer patients is low compared to lung, colorectal, liver, and stomach cancer patients [2], the breast cancer burden is costly and an enormous obstacle to increasing the quality of life for women and girls around the world [1].

One of the key approaches to breast cancer management and control is cost-effective screening and early detection [3]. Although mammography is a widely used breast cancer screening technology, it has technical, logistic, and diagnostic limitations with respect to false positives and negatives, convenience and participation, limitations with younger women and dense breasts, exposure to radiation, and the limitation of detecting tumours of small sizes in the early stage of breast cancer [4,5]. Therefore, alternative tools for the screening and detection of breast cancer are urgently needed.

Over the past decade, there has been a surge of interest in the study of metabolite, protein, and lipid biomarkers derived from high-throughput molecular profiling of various biofluids of patients with breast cancer, such as blood (serum/plasma) [6,7,8], urine [9,10,11], saliva [12,13], ductal lavage fluid [14], nipple aspiration fluid [14,15,16], and tears [17,18]. While increasing evidence from numerous studies corroborates the utility of non-nucleotide biomarkers in diagnosing breast cancer, there have often been inconsistencies in biomarkers reported by different studies. One of the major contributors to the observed inconsistencies is the lack of gold-standard methods and protocols across different phases of biomarker discovery and laboratory testing, including the pre-analytical phase (specimen collection and sample processing), analytical phase (measurement of analytes), and post-analytical phase (data pre-processing, statistical analysis, model development, interpretation of results, and reporting) [19,20].

There are several factors across each of these phases which can impact the outcomes and the reproducibility of the findings, that is, the ability to replicate the same results for the same condition [21]. It is, therefore, essential to review diverse approaches commonly practised in each phase of liquid biopsy biomarker discovery to help research and industry sectors to adopt the best practices and standardise their pipelines. There has been a dearth of comprehensive reviews on experimental and methodological variations from the pre-analytical to post-analytical phases of liquid biopsies for breast cancer diagnosis and beyond. Furthermore, while former studies have reviewed liquid biopsies in breast cancer [22,23,24,25], the focus has often been on circulating tumour cells (CTCs) or nucleotide-based biomarkers (e.g., circulating tumour DNA (ctDNA), circulating or extracellular vesicle-encapsulated microRNA, and platelet-derived RNAs), with non-nucleotide biomarkers often limited to proteins. Therefore, non-nucleotide-based circulating biomarkers have been reviewed to a much less extent, demanding focused attention given the differences in the pre-analytical and analytical phases and diversity of techniques of molecular profiling for non-nucleotide compared to nucleotide-based analytes.

**Figure 1:**
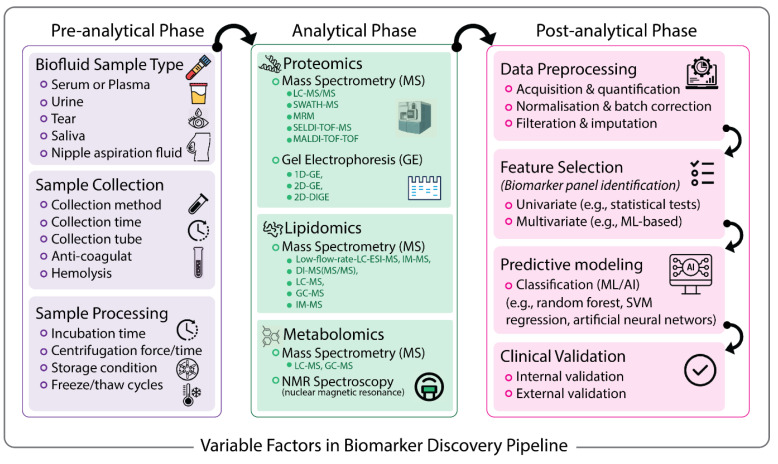
Variable factors involved in biomarker discovery pipeline. This schematic flow chart illustrates the influential factors involved in typical pre-analytical, analytical, and post-analytical stages in proteomics, metabolomics, and lipidomic investigations in breast cancer liquid biopsy.

To address this resource gap, we conducted a comprehensive search of the literature published in the last two decades (2001–2023), focusing on high-throughput omics approaches in breast cancer liquid biopsy using Google Scholar, PubMed, Elsevier, and Scopus databases to query a combination of medical subject headings (MeSH) and terms including breast cancer, proteomics, metabolomics, lipidomics, liquid biopsy, plasma, serum, blood, urine, saliva, tear, nipple aspiration fluid, and ductal lavage fluid. We extracted over 200 relevant public shed articles (Supplementary Table S1), reviewed their biomarker discovery pipelines, and investigated the trend in the target biofluids and biomarker types. Figure 1 illustrates different factors affecting biomarker discovery outcomes from the pre-analytical to post-analytical phase, according to the findings of previous studies. More details on exemplar studies are summarised in Table 1 (and Supplementary Table S2) to showcase the diversity of protocols adopted across different phases of biomarker discovery, focusing on circulating non-nucleotide-based breast cancer diagnostic makers (lipids, proteins, and metabolites), highlighting the demand for standardised procedures.

## 2. Pre-Analytical Variables

In biomarker discovery studies, body fluid sources, sample collection procedures, handling, preparation steps, and storage conditions are defined as pre-analytical variables [42,43]. They are one of the most error-prone, time-consuming, and laborious steps in biomarker identification, and they affect the sensitivity, reproducibility, and selectivity of analysis and need to be carefully considered during the project design [44]. In the following subsections, we will outline the intricacy of the pre-analytical phase and its significance for biomarker discovery.

### 2.1. Biofluids Are Excellent Sources of Biomarkers

Recently, different types of body fluids have obtained great attention as sources of biomarkers for the detection and monitoring of breast cancer due to their low complexity and simpler sample collection and processing procedures compared to solid tissues, sustainable accessibility, and the ability to be measured repeatably in a minimally invasive way [45]. The major challenge in biomarker discovery from body fluids is the identification of biomarkers specific to the type of cancer. For example, a proteomic analysis of five different body fluids by Zhao et al. suggested that the proteome of body fluids may indicate the holistic functions of the whole body rather than that of adjacent tissues [46]. Therefore, the identification of biomarkers released into the body fluid by cancerous lesions may be difficult. Nonetheless, the metabolic changes that occur in the body due to the onset of cancer can be reflected in the metabolic/proteomic profile of body fluids. Furthermore, daily water intake or microbiome profile may alter the protein or metabolite concentration in a patient’s body fluid [47,48] and the biomarker concentration may depend on the sample collection method. Thus, the pre-analytical phase of biomarker discovery workflows should be stringently standardised.

The selection of appropriate body fluids depends on the type of omics study (i.e., proteomics, lipidomics, or metabolomics), as one specimen may be advantageous over the other. For example, urine samples, mainly composed of metabolites and end products of biochemical reactions, are more suitable for metabolomic analysis [49]. Furthermore, compared to saliva, which comprises 99% water and 0.3% protein, serum and plasma are more appropriate for proteomic investigations [50]. In the following sections, we discuss the commonly used biofluids for biomarker discovery.

#### 2.1.1. Serum and Plasma

Blood is believed to have the most complex human-derived circulating biomarkers and therefore has attracted considerable research attention. So far, over 12,000 proteins, 600 lipids, and 300 metabolites have been profiled from blood samples [51,52,53], and the concentrations of many circulating analytes were found to be different in plasma and serum [54,55,56]. For example, Liu et al. revealed that some metabolites, including most amino acids, hypoxanthine, carbohydrates, b-hydroxybutyrate, and glycerol-3-phosphate, were significantly lower in plasma compared to serum. In contrast, other metabolic products such as citrate, fumarate, pyruvate, glycerate, nitrogen metabolites, urate, and hydroxylamine were significantly higher in the plasma [54]. Furthermore, studies indicate that the total concentration of several lipids, including triglycerides (TGs), phosphatidylcholines (PCs), and HDL cholesterol, were higher in serum than in EDTA or citrate plasma [57,58].

Breier et al. reported that the reliability of metabolite measurements was slightly higher in serum samples compared to plasma [59]. The reason for this may be the higher metabolite concentration in serum compared to plasma, which provides greater sensitivity for biomarker identification [60,61]. However, the concentration of some metabolites involved in platelet aggregation will be different from their actual level in serum as the clotting process causes these metabolite levels to increase. Therefore, such metabolites will need to be measured from plasma [58]. In the study by Ishikawa et al., it has been demonstrated that plasma is more suitable than serum for studying lipid biomarkers because the clotting process was found to affect serum lipid levels [62]. Moreover, lipids showed the lowest biological variation in plasma citrate samples, implying the suitability of plasma for quantitative targeted lipidomics [60]. Nonetheless, the method and conditions by which the plasma was prepared need to be standardised to avoid detecting differences due to the time used, temperature or type of tubes, centrifuge used, or how the sample is stored (e.g., 4 °C −20 °C, −80 °C, or snap frozen).

When the blood clot is removed during serum preparation, the concentration of high-abundance circulating proteins, such as fibrinogen, will significantly decrease in serum, making it much easier to detect low-abundance proteins. At the same time, some proteins are released from the platelet during the blood coagulation process. This phenomenon can vary sample-to-sample and may lead to the false positive identification of protein biomarkers from serum [63,64]. A study by Tammen et al. suggested citrate plasma or platelet-depleted EDTA plasma for studying the low-molecular-weight proteome [65]. In 2005, the HUPO’s Human Plasma Proteome Project (HPPP) recommended using EDTA plasma as the preferred sample for all proteomic analyses [66]. Therefore, it is not possible to measure the biomarkers of interest from plasma and serum interchangeably. Based on the aims of the study and the target biomarker, either plasma or serum may need to be chosen.

**Figure 2:**
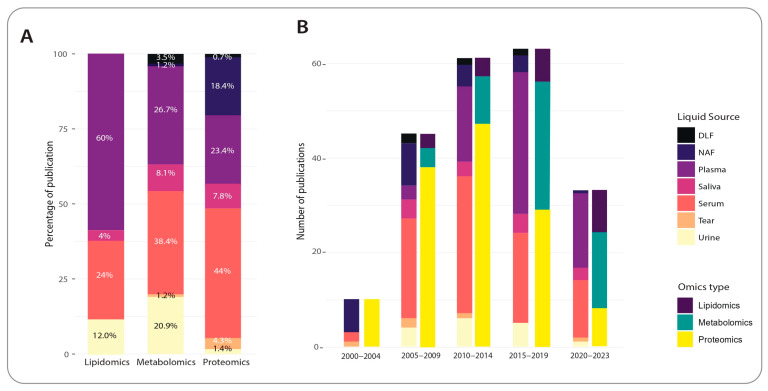
The proportion of metabolomic, lipidomic, and proteomic investigations carried out on various biofluid samples of breast cancer between January 2001 and April 2023. (**A**) Percentage of metabolomic, lipidomic, and proteomic studies according to their biofluid source. (**B**) The trend in studying non-nucleotide-based biomarkers from various biofluids of breast cancer in the last twenty years.

As shown in Figure 2, a greater tendency to use serum over plasma has not been observed in breast cancer metabolomics investigations. The number of metabolomics studies that used serum as the biofluid sample of choice was relatively similar to those that utilised plasma samples. However, plasma was the preferred matrix over serum for breast cancer lipidomic investigations, with approximately 60% of the publications reporting on plasma as opposed to approximately 24% reporting on serum. In contrast, serum samples were used in approximately 44% of the studies focusing on proteomics investigations of breast cancer, which is much higher than plasma selection.

#### 2.1.2. Urine

Urine is one of the most widely used human body fluids for routine testing due to its less complex composition [67,68]. Many studies on urine biomarkers for breast cancer screening and diagnosis are still in the discovery phase; hence, further cohort investigations are needed to validate their sensitivity and specificity [22].

There are several types of urine collection approaches, including random, first-morning, second-morning, and 24-h collections [69]. Each kind has unique advantages and disadvantages for metabolomic, proteomic, and lipidomic investigations. Although a random urine sample is presumably the most straightforward collection approach, it is rarely the preferred choice, as depending on the collection time, urine may be excessively diluted due to water intake, and the patient’s diet and exercise would have affected its composition [68,70]. The first-morning urine sample is generally considered appropriate for proteomic studies because it contains the largest amount of total proteins [71] and shows the lowest variation compared to the 24-h urine samples [68,72]. Conversely, the midstream second-morning urine collected after an overnight fast is recommended for metabolomic profiling, as the pattern of metabolites in the first-morning urine may reflect nutrients consumed the day before [69,73]. Although urine collection time is a critical factor, it has been neglected by many studies focused on urinary metabolomics in breast cancer [74,75,76]. However, in a few investigations, it has been indicated that first-morning urine collection was used [77,78].

In terms of lipidomic analysis, there is a lack of information demonstrating the characteristics of each urine sample type based on the time of sampling for lipid biomarker discovery. Furthermore, few investigations have been performed on urinary lipidomics in patients with breast cancer, in which the detailed information of urine collection protocols has not been addressed well [79,80].

Another aspect to consider using urine as the source of biomarkers is the difference in the microbiome composition of the urinary tract and the vaginal tract in women. Due to the microbiome–host interaction, the results can be affected. The microbiota may produce and secrete proteins, lipids, etc., which may confound the biomarker discovery and may also metabolise the host-secreted biomarkers in the sample. It has been shown that urinary microbiota composition differs by menopausal status in patients with breast cancer [81]. Moreover, regardless of menopausal status, cancerous patients had increased levels of Gram-positive bacteria, including *Corynebacterium*, *Staphylococcus*, *Actinomyces*, and *Propionibacteriaceae* [81], which may influence the metabolite and protein content of urine.

#### 2.1.3. Tears

The tear’s composition, especially proteins, can be substantially affected due to the sample collection procedure [82,83,84]. Schirmer’s test strips (STSs) and microcapillary tubes (MCTs) are the most popular tear sampling procedures [85]. Pieragostino et al. [86] reviewed the advantages and disadvantages of collection techniques previously. STSs have been used in most proteomics studies in breast cancer so far [17,18,87]. Results from the analysis by Nättinen et al. [83] indicated that Schirmer strip samples had a ten-fold greater mean total protein content compared to MCTs. To date, there is no agreement on how the tear sampling procedures impact the proteomic data. Sample handling, such as strip cutting, has been shown to increase the risk of contamination and protein loss, making the results even more variable [84]. Therefore, the most appropriate and reliable tear sampling approaches are needed for the accurate and repeatable detection of tear biomarkers.

#### 2.1.4. Nipple Aspiration Fluid

Nipple aspirate fluid (NAF) in non-lactating women is a fluid secreted by breast epithelial duct cells and can be collected with various degrees of effectiveness, ranging from 34% to 90% by utilising a milk-expressing pump, nasal oxytocin spray, and gentle breast massage [31,88,89,90]. Proteins are the main components of NAF, with concentrations up to 170 mg/mL, which can be more than that found in plasma [91]. However, there are some challenges when using NAF as a source of protein biomarkers. Firstly, NAF droplets may not be acquired from the duct where carcinogenesis has occurred [92]. Furthermore, it has been shown that the colour and viscosity of NAF can affect biomarker identification when using spectrophotometry approaches [92]. Li et al. proposed that the notable differences in the results spectra between NAF samples in a group may stem from several reasons, including the biological variation in the breast duct’s microenvironment and variability of the protein concentration in the samples (equal sample volume was examined rather than equal protein concentration) [14]. Given these variations and challenges in NAF sample examinations, it is difficult to cross-compare the findings of different investigations. Another challenge in using NAF is that the microbiome profile and host–microbiome interaction may interfere with the biomarker studies. The study by Chan et al. showed that the microbiota composition of NAF significantly differs in patients with breast cancer compared to healthy women, which may affect the multi-omics profile of NAF [93] for biomarker discovery.

#### 2.1.5. Saliva

The safe, non-invasive, and repeatable collection makes saliva a good target for biomarker discovery. Investigations showed significant differences in the level of metabolites in saliva that can be used as biomarkers for breast cancer diagnosis [37,94,95]. However, the exceptionally diverse composition of saliva arising from age, diet, gender, and time of day of the collection makes it a challenging choice of biofluid for biomarker studies.

Protein degradation is one of the main reasons for the irreproducibility of salivary proteomic analyses. The proteolytic degradation commences just as the proteins enter the oral cavity and continues post-collection of salivary samples, leading to substantial differences in biomarker profiles [70]. Furthermore, salivary biomarkers can be affected by the site of collection. For example, Cui et al. showed that the concentration of several metabolites was different in whole saliva, parotid saliva, and submandibular/sublingual saliva [96]. Moreover, Assad et al. propounded that small variations in the collection and storage procedure affect the free amino acid content of saliva as it comprises proteinases and peptidases [97], resulting in irreproducible results between studies.

#### 2.1.6. Extracellular Vesicles

Extracellular vesicles (EVs) are rich sources of circulating biomarkers in blood that have been of interest in many recent studies, with demonstrated utility in breast cancer diagnosis, as reviewed previously [98]. Continuous production, release, and uptake of existing EVs by different types of blood cells, as well as the delay between blood collection and preparation of plasma or serum, need to be considered when EVs are used for biomarker discovery [99,100]. It is shown that physical activity undertaken prior to sample collection, besides other pre-analytical parameters such as collection tube, centrifugation, and storage time, may influence morphology, size, and stability, as well as the downstream characterisation of EVs [101,102]. EV isolation and enrichment are other discriminatory pre-analytical factors in many studies, as there is no established gold-standard protocol to purify and isolate EVs. For instance, centrifugation is one of the main parameters that impact the reproducibility of EV isolation and purification [102,103]. This may complicate cross-comparison between studies as well as the external validation of biomarkers. This lack of standardised guidelines in EV research has triggered international efforts and consortiums, such as EV-TRACK (https://evtrack.org/index.php, accessed on 2 May 2023), to facilitate the standardisation of EV research through increased systematic reporting [104].

Based on the study published in 2020 [105], the concentration and size of the microvesicles (MVs), which are a sub-type of EVs, differ in plasma and serum. While MVs have lower concentrations in serum, small-sized MVs are higher in serum than large-sized MVs. In another study by Palviainen et al., the protein profiles of plasma EVs were different between serum and plasma [106]. In order to reduce vesicle release from blood cells, most procedures suggest using plasma rather than serum [101]. EVs and MVs in cancer biomarker discovery have previously been reviewed in detail [99,101,107,108]. In breast cancer studies focusing on EVs, plasma was used as the main source compared to serum [108], regardless of the type of EV composition.

### 2.2. Sample Collection and Processing Variables Impact the Discovery of Accurate Biomarkers

In addition to biofluid type, other pre-analytical variables, including anti-coagulants, collection tubes, incubation times (pre-centrifugation processing delay), storage time and temperature, and freeze–thaw cycles, can also influence biomarker levels, thereby affecting the analytical reproducibility [42,43,109]. Some distinguished influential variables that may occur during sample collection and handling are presented in Table 2 to highlight the importance of considering these facets in prospective proteomic, metabolomic, and lipidomic studies. The information presented in Table 2 reiterates that the pre-analytical phase should be meticulously controlled and regulated to prevent unfavourable impacts on biomarker discovery and underscores the need for highly standardised protocols.

### 2.3. Trends in Non-Invasive, Non-Nucleotide Biomarker Discovery for Breast Cancer

As discussed above, in biomarker discovery studies, the ease of sample collection, reproducibility, and effective variables are some of the critical factors. Biomarker investigations for the detection and prognosis of breast cancer are more concentrated on non-invasive approaches rather than a tissue biopsy. Figure 2 illustrates the proportion of metabolomic, lipidomic, and proteomic investigations carried out on various biofluid samples of breast cancer between January 2001 and April 2023. It demonstrates that the number of studies exploiting non-nucleotide-based biomarkers from various biofluids has increased in the last ten years. Although proteomics has dominated the field for many years, there has been a shift to metabolomics and lipidomics since 2015. Regarding biofluid sources, although various biofluids have been exploited for biomarker discovery, blood continues to be the primary biofluid for biomarker discovery (plasma and serum). Notably, serum was the primary source before 2015, and plasma was the primary source from 2015 to 2019. The preference for choosing blood over other biofluids might be due to the fact that, compared to the other biofluids, fewer variables, including exposure to air, possible effects of their microbiome on the abundance and composition of analytes, time of collection, and the high proportion analytes related to adjacent tissues may affect the study outcomes [137,138,139,140].

Furthermore, protocols and analysis pipelines of plasma and serum may be more standardised compared to other biofluids. Proteomics and metabolomics are emerging fields that have expanded rapidly as a result of parallel improvements in bioanalytical platforms and methods for data analysis [141]. As shown in Figure 2, the trend of research using proteomics to identify biomarkers has been overtaken by lipidomics and metabolomics in more recent years. This may be due to the development of new protocols and methods for metabolome and lipidome purification, advances in analytical techniques, and awareness of their potential use for biomarker discovery.

## 3. Analytical Techniques for Biomarker Discovery

Apart from the pre-analytical variables, the wide dynamic ranges, sensitivity, and specificity of analytical methods are major challenges in biomarkers discovery, which can affect the reproducibility of biomarker identification. For example, because some biomarkers have a very low abundance in the selected biofluid, the sensitivity of the analytical method can limit the number of discovered proteins [70]. Detailed information on commonly used techniques in proteomic, metabolomic, and lipidomic investigations is included in Table 3 and summarised below.

### 3.1. Proteomic Approaches

Proteomic workflows can be categorised as gel-based and gel-free methods coupled with array-based and mass spectrometry-based (MS) techniques [159]. Mass-spectrometry (MS) is the most commonly used approach in proteomic studies of breast cancer [160]. Time-of-flight, triple quadrupole, and orbitrap mass spectrometers can be coupled with different ionisation procedures, including surface-enhanced laser desorption/ionisation (SELDI), matrix-assisted laser desorption/ionisation (MALDI), and electrospray ionisation (ESI) for proteomic applications [160]. Although most of the investigations utilised the SELDI-TOF-MS method for breast cancer diagnosis as a potential discovery method, the reproducibility was questionable due to the low resolution of SELDI-TOF-MS data and chip-to-chip variation. In contrast, MALDI-TOF-MS shows higher reliability and robustness and is favoured in clinical proteomics [161]. However, it is not without limitations; for example, MALDI-TOF-MS is sensitive to impurities such as salt, causing problems with the reproducibility of the results [68].

Two-dimensional gel electrophoresis (2-DE) is a technique widely used in qualitative proteomic investigations of breast cancer [10,28]. However, this technique has some drawbacks, including weak inter-assay reproducibility, low sensitivity for the detection of proteins with either very low PH (<3) or high PH (>10) values, and too small (<10 kD) or too large (>150 kD) molecular masses, as well as the inability to identify hydrophobic and low abundant proteins [162]. In contrast, the two-dimensional difference in the gel electrophoresis (2D-DIGE) approach has demonstrated higher sensitivity and improved reproducibility [155].

Other factors, such as diversity in binding/washing buffer conditions and the chemistry of ProteinChip surfaces, can influence the binding and identification of various proteins, leading to discrepancies in biomarker discovery [27]. For example, IMAC3 (Immobilized Metal Affinity Capture) chips capture proteins via chelation of metal ions, whereas H4 chips absorb by hydrophobic interaction; consequently, the proteins captured by these chips are distinct and would lead to irreproducible results [163,164]. Therefore, analytical procedures should be standardised among research and clinical laboratories for a precise interpretation and interlaboratory comparison of data.

### 3.2. Metabolomic Approaches

Two main analytical techniques are commonly employed in metabolomic investigations: mass spectrometry and nuclear magnetic resonance (NMR) spectroscopy [32]. Although NMR has the capability to measure metabolites with high reproducibility in complex samples without the need for pre-preparation of biological fluids, it shows low sensitivity [165]. Mass spectrometry techniques used for breast cancer studies include ultra-high performance liquid chromatography coupled with quadrupole time-of-flight (UPLC-QTOF-MS) [166,167], gas chromatography-mass spectrometry (GC-MS) [168,169,170], liquid chromatography-mass spectrometry (LC-MS) [8,171], and ultra-fast liquid chromatography-tandem mass spectrometry (UFLC-MS/MS) [172]. However, the LC-MS and GC-MS methods have been frequently applied for biofluids [173]. LC-MS stands as the most suitable approach for the sensitive identification of biomolecules with high reproducibility [174], while GC-MS shows relatively stronger chromatography with distinct peak separation [175].

### 3.3. Lipidomic Approaches

Technological advancements in liquid chromatography, high-resolution accurate mass spectrometry, and NMR spectroscopy have improved the high throughput analysis of lipid molecules [176]. Many mass-spectrometry-based approaches are used in lipidomic studies, each with unique characteristics, advantages, and disadvantages [177]. Mass spectrometry imaging (MSI), direct infusion or shotgun MS, and MS accompanied by initial chromatographic separation such as GC, LC, and thin-layer chromatography (TLC) are the main three infrastructures of lipidomic investigations [147]. Shotgun MS, in which the analyte is not separated by prior chromatography, performs poorly in detecting less-ionisable and low-abundant lipids due to ion suppression, during which the signals stemming from weakly ionised lipid species are buried in the signal of strongly ionised lipids [178,179]. However, the detection of such lipids can be improved by a pre-separation approach, such as LC-MS, which has demonstrated high sensitivity, specificity, and remarkable separation efficiency for lipids [147].

## 4. Post-Analytical Steps and Variations

### 4.1. Data Pre-Processing

Mass spectrometry-based techniques have become the mainstream methods for high-throughput and unbiased proteomics, metabolomics, and lipidomics profiling. Several forms of proprietary and open-source software have been developed for data acquisition and quantification, as discussed elsewhere [180,181]. These tools have different underlying assumptions and algorithms for searching (e.g., database vs. de novo) and molecular species quantification [182], which contributes to the discrepancy of generated data across different studies. A comprehensive benchmarking is required to compare data acquisition and quantification techniques and to provide a guideline for the best practices.

Once quantified, high-throughput spectrometry or spectroscopy data are often subject to multiple pre-processing steps to stabilise variance, reduce systematic bias or technical variations, and impute missing data. The choice of pre-processing approach can substantially affect the data quality and validity of downstream analyses. For instance, Mertens [183] argued in favour of log-transformation to mitigate the skewness and standardise spectrometry data, which has raised concerns regarding using so-called “closure normalisation”, e.g., data normalised by the sum of the combined expression in exerting spurious biases in the correlations between the spectral measures masking true population associations. Nonetheless, the diversity of the available pre-processing statistical approaches demands benchmarking studies to systematically investigate their effect on the quality of data and the reproducibility of the biomarkers identified. Välikangas et al. [184], for instance, evaluated normalisation methods in quantitative label-free proteomics and demonstrated the variations in outcomes of downstream analyses (e.g., differential expression) depending on the choice of the normalisation method. Despite the importance of pre-processing, we frequently observed unclear and incomplete descriptions of the approaches undertaken in the literature we have reviewed in relation to the non-nucleotide biomarkers of breast cancer (Table 1 and Supplementary Table S2).

### 4.2. Biomarker Signature Panel Identification (Feature Selection)

From the computational perspective, signature panel identification can be formulated as a feature selection or extraction problem, which implies the *selection* of a set of molecules (e.g., proteins, lipids, or metabolites) that best stratify the groups of interest (e.g., cancer vs. control) or the extraction of latent features from the entire omics profile (e.g., embeddings derived via dimensionality reduction). Feature selection has been historically performed via differential analysis (i.e., statistical hypothesis tests such as *t*-test or Mann–Whitney U test). However, while differential analysis can detect functionally relevant molecules, it is ineffective in selecting features with optimal predictive power [185] as it is a univariate approach overlooking nonlinear relationships among multiple biomarkers, whose collective effect contributes to the prediction of a phenotype, disease outcome, or treatment response. Several sophisticated machine learning-based methods have been developed by the computer science community for feature extraction or selection of predictive variables from high-dimensional data, which can substantially enhance signature panel identification, and the development of predictive models and cancer diagnostics as previously benchmarked [186]. Despite the proven utility of machine learning and nonlinear, multivariate feature selection in identifying biomarker signatures with high sensitivity and specificity, statistical hypotheses testing has been the dominant approach adopted in non-nucleotide breast cancer biomarker discovery, as outlined in Supplementary Table S2.

### 4.3. Biomarker Predictive Modelling (Classification)

After feature selection (or extraction), the identified biomarker signature panel can be used as predictive variables of a classifier algorithm to stratify patients into categories of interest (e.g., cancer vs. normal). A classifier algorithm often implements a mathematical function that maps input data to a category upon learning from a training cohort. Different classifiers have been implemented as multi-variate cancer diagnostics models, including commonly used algorithms such as random forest, support vector machines, logistic regression, artificial neural networks, and ensemble approaches (i.e., predictive models composed of a weighted combination of multiple classifiers) [187]. For a long time, improving the prediction accuracy has been the primary focus of biomarker discovery predictive modelling. However, biomarker discovery methods should be assessed based on prediction accuracy as well as robustness, defined as the generalisability of the model to diverse cohorts. In recent years, the stability of biomarker discovery has gained more attention, as reviewed previously [188]. Nonetheless, in breast cancer liquid biopsy studies, the adoption of classifiers as diagnostic models has been limited (Supplementary Table S2), contributing to the lack of highly predictive and robust diagnostic tests.

### 4.4. Clinical Validation

Extensive validation is necessary before the clinical implementation of a diagnostic test. Validation of a predictive model using the dataset at hand (referred to as the development dataset) is often referred to as an internal validation, wherein the dataset is divided into the test and train sets, using the latter for model development and optimisation and the former for model validation. In addition, to mitigate model overfitting, particularly in small datasets, data re-sampling techniques, such as bootstrapping or cross-validation, can be used to account for the selection bias and to quantify the stability of the predictive performance [189].

Based on our literature review, the majority of breast cancer liquid biopsy studies have only reported the prediction performance of biomarkers upon internal validation, which is not sufficient to confirm model generalisability. In order to progress towards implementation and technology readiness, extensive external validation is required, wherein the model’s predictive performance is quantified using data collected from participant cohorts external, temporally and/or geographically, to the development dataset [189].

Besides the validation of the prediction models, the analytical parameters should be optimised, followed by the validation of the parameters according to regulatory guidelines [190,191]. The clinical performance of the test should then be compared to the gold-standard method, e.g., mammography [192,193]. When the technology is implemented, prospective clinical studies should be conducted to assess if the assay improves patient outcomes and reduces healthcare costs [192,194].

## 5. Conclusions and Future Perspective

Our major biofluid biomarker discovery pathway throughout the last decades was focused mainly on nucleotide-based biomarkers for early breast cancer diagnosis. However, in recent years, the investigation of proteomics, lipidomics, metabolomics, and microbiome profiles, along with EV cargo, has been increased to introduce new biomarker profiles, not only for blood but also for other types of body fluids, as we have comprehensively reviewed here. We also reviewed the effect of different procedures, from sample collection and processing to data analysis and validation. The lack of standard protocols in different parts of biomarker discovery can be a key factor hindering the clinical implementation and manufacturing of commercialisable assays or clinical tests. Therefore, one of the future efforts in breast cancer biomarker studies is to standardise the liquid biopsy assay procedures and analysis platforms. This will give a better opportunity to combine and compare results from different studies and develop breast cancer liquid biopsy consortiums to advance and validate liquid biopsy technologies, homogenise guidelines, and standardise data for the development of breast cancer biomarkers. Some initiatives have already been implemented by the National Institute of Health (https://prevention.cancer.gov/major-programs/liquid-biopsy-consortium, accessed on 2 May 2023), targeted for early-stage cancer detection on a wide range of cancer types.

Due to the ongoing advances in non-invasive biomarker discovery, technology, and data analytics, the future of the field is moving towards multi-omics liquid biopsy and non-invasive blood tests (or other bodily fluids) through the simultaneous assessment of different omics data (e.g., genomics, transcriptomics, and proteomics) from body fluids for cancer detection and monitoring. Multi-omics approaches could provide complementary information on the presence of the dysregulated bodily processes leading to disease, enabling early detection of tumours, and they have demonstrated utility in enhancing the sensitivity and specificity of cancer detection as we construct a fuller picture [195]. Despite its advantages, multi-omics liquid biopsy is facing slow adoption and implementation. So far, there have been limited studies using this approach for breast cancer identification emerging over the last few years (Supplementary Table S1). One major obstacle is limited sample availability and/or technical difficulties associated with generating complete multi-omics datasets due to the uneven maturity of different omics approaches. Moreover, the growing gap between generating large volumes of data compared to data processing capacity and available integrated datasets are of concern. Additional efforts are needed for the standardisation of multi-omics operational procedures and data integration, from robust pre-processing and operational guidelines to data integration and validation.

## Figures and Tables

**Table 1:** Protocol variability in breast cancer-associated biomarker discovery workflows.

Aim	Pre-Analytical Phase						Analytical Phase		Post-Analytical Phase	Ref
	BioSource	Collection Tube	Time to Sample Processing	Centrifugation	Storage	Tumour Grade	Technique	Validation Method	Hypothesis Test Performed	
Proteomic	Serum	NA	4 °C for 1–2 h	3000 rpm for 5 min + 12,000 rpm for 5 min	−80 °C	NA	SELDI-TOF-MS	SDS-PAGE MALDI-TOF/TOF	*t*-test ANOVA	[26]
	Serum	Plastic tube with clot activator	15 min	3280× *g* for 5 min, 4 °C	−80 °C	NA	SELDI-TOF MALDI-TOF-TOF	NA	*t*-tests Multivariate discrimination analysis ANOVA ANN ROC	[27]
	Plasma	K2EDTA tube	2 h	1300× *g* for 10 min	−80 °C	NA	1D gel electrophoresis 2D gel electrophoresis LC-MS/MS	WB	Unpaired *t*-test	[28]
	Plasma	EDTA tube	30 min	4000× *g* for 30 min	−80 °C	NA	LC-MS/MS	WB	*t*-test	[29]
	Plasma	Sodium EDTA tube	NA	1400× *g* for 5 min, 4 °C	ND	Low and high grade	Label-free nano-LC/MSMS	WB	Mann–Whitney	[30]
	NAF	Graduated micropipette	Immediately	1500 rpm for 10 min	−80 °C	I/II	SELDI-TOF-MS	ELISA	Supervised and unsupervised cluster analysis	[14]
	NAF	Tube pre-treated with cocktail mixture of protease inhibitor	<30 min	NA	ST: −20 °C LT: −80 °C	I–III	1D LC-MS/MS	NA	Pearson’s correlation coefficients Paired Student *t*-test	[31]
	Urine	Sterile tube	Immediately	2000× *g* for 10 min, 4 °C	ST: −20 °C LT: −80 °C	II–III	Label-free LC-MS/MS	WB	ANOVA	[9]
	First Morning Urine	Tube containing 0.02% *w*/*v* Sodium Azide)	NA	NA	ND	I/II	Standardisation phase: 2D gel electrophoresis Discovery phase: 2D-DIGE, MALDI-TOF-TOF, SWATH-MS, iTRAQ, LC-QTOF	WB MRM	Supervised and unsupervised cluster analysis Multivariate analysis Chi-square	[10]
Metabolomic	Plasma	EDTA tube	<2 h	3000× *g* for 10 min, 4 °C	−80 °C	I–III	LC-MS	NA	Kruskal–Wallis Mann–Whitney U test ROC	[32]
	Plasma	K2EDTA tube	Immediately	1500× *g* for 10 min, RT	−80 °C	I–III	LC-QTOF-MS LC-QQQ-MS	NA	Student’s *t*-test PLS-DA OPLS-DA	[33]
	Serum	Vacutainer tube	30 min	3000 rpm for 10 min, 4 °C	−80 °C	I–III	UHPLC-QTOF-(ESIþ)-MS	NA	Pearson ROC PCA PLS-DA *t*-test	[34]
	First Morning Urine	NA	NA	3000× *g* for 10 min, RT	−80 °C	I/III	GC–MS LC-QTOF/MS	NA	PCA OPLS-DA Univariate analysis Unpaired *t*-test Mann–Whitney U test	[35]
	Saliva	Polypropylene tube	NA	NA	−80 °C	0–IV	CE-TOF-MS	LC-QQQ-MS	Mann–Whitney U test Kruskal–Wallis Multiple logistic regression Multiple AD tree models	[36]
	Saliva	NA	10 min	13,500 rpm for 20 min, 4 °C	−40 °C	I–IV	HILIC-ESI-MS RPLC-ESI-MS	NA	Mann–Whitney U test PLS-DA PCA	[37]
Lipidomic	Plasma	Heparin tube	NA	1500× *g* for 15 min	−80 °C	I/II	UPLC-QTOF/MS	NA	*t*-test One-way ANOVA OPLS-D	[38]
	Plasma	EDTA tube	<2 h	2600× *g* for 10 min, 4 °C	−80 °C	0- II	LC-ESI-MS/MS	NA	*t*-test Binary logical regression ROC	[39]
	Serum	NA	NA	NA	−80 °C	NA	NMR spectroscopy	NA	*t*-test Mann–Whitney U test Chi-square Binary logistic regression	[40]
	First Morning Urine	NA	NA	3000× *g* for 10 min, RT	−80 °C	I/III	LC–MS	NA	OPLS-DA Univariate analysis Unpaired *t*-test Mann–Whitney U test ROC	[35]
	Saliva	Polypropylene tube	NA	10,000× *g* for 10 min	Without freezing and storage	I–III	IR spectroscopy	NA	Mann–Whitney U-test Kruskal–Wallis Multivariate comparison	[41]

NA: Not Available, EDTA: Ethylenediaminetetraacetic Acid, K2 EDTA: Dipotassium Ethylenediaminetetraacetic Acid, h: Hour, min: Minute, RT: Room Temperature, ST: Short-Term, LT: Long-Term, WB: Western Blotting, ELISA: Enzyme-linked Immunosorbent Assay, ANN: Artificial Neural Network, ROC: Receiver Operating Characteristic.

**Table 2:** Pre-analytical variables during sample collection and handling.

Pre-Analytical Variable	Literature Findings
Collection Tubes	Sample collection containers are frequently overlooked variables in laboratory settings [110] The same sample might have different protein profiles when collected in two different types of tube [111,112,113] Blood tube components may adsorb some analytes, particularly proteins, leading to their detection loss [114] Release of plasticisers from tubes into samples may adversely affect high-resolution mass spectrometric examinations [62]
Anti-Coagulant	For metabolomics profiling, sodium fluoride (NaF) and EDTA salts caused less interference than sodium citrate or lithium-heparin [115] Heparin [116,117] or EDTA plasma [115,118,119] is recommended for mass spectrometry-based lipidomic and metabolomic analyses; EDTA plasma is unsuitable for NMR-based approaches as it leads to interferences in the spectra [120] EDTA anti-coagulant is preferable for proteomics [111,121]
Hemolysis	One of the most common pre-analytical errors [122] Destruction of red blood cells Release of proteins, metabolites, and lipids into serum or plasma [115,123] May obstruct correct profile interpretation [115,122] MS-based assessments may be affected [124]
Incubation Time	Many chemical and enzymatic reactions will continue and eventually metabolise the lipids [125,126] Blood cells constantly release, uptake, and metabolise compounds [69,119,127] Metabolites are more sensitive to prolonged incubation at room temperature than at 0–4 °C [124] Peptides and degraded proteins can be released from blood cells [128]
Centrifugation Force	Minor differences in centrifugation could lead to variations in metabolomic patterns [129] Higher centrifugation (between 2300 and 4000× *g* for 5–10 min) is recommended for lipidomic and metabolomic studies [69] Centrifugation at 1300–2000× *g* for 15 min was recommended for proteomic studies [130]
Storage Conditions	Several analytes can be affected by storage temperature and time [131] Serum proteins change more at room temperature compared with −20 °C and −80 °C [132] Storage at lower temperatures, such as −80 °C, is recommended [133,134]
Freeze–Thaw Cycles	Repeated freeze–thaw of samples can result in profile alterations [135,136] One freeze–thaw cycle leads to dramatic alterations in several urinary proteins [132]

**Table 3:** Advantages and disadvantages of various analytical techniques used for proteins, metabolites, and lipidomics biomarkers.

Techniques	Advantages	Limitations	Biomarker Type
MALDI-TOF-MS [142,143,144,145]	Rapid and straightforward operability Low sample volumes Mostly single-charged registered ions [M-H]+ High throughput High accuracy, resolution, and sensitivity No staining, labelling, anti-body, and hybridisation Suitable for large polypeptides (>30 kDa) detection	Variation in the surface of the MALDI-TOF target Limited dynamic range Sensitive to contaminants Low reproducibility	Proteins
SELDI-TOF-MS [146]	High throughput Low sample volumes High sensitivity Easy operability Suitable for small peptides (∼500 Da) detection Suitable for low MW, modified, truncated, or fragmented proteins detection	Failure of the validation process Low reproducibility Low resolution Biased toward smaller peptides and proteins (<30 kDa) Problems in larger MW proteins and PTM identification Ion suppression Prone to artefacts generation	Proteins
LC-MS [68,147,148,149,150]	High throughput High resolution Suitable for low and high-molecular-weight compounds High sensitivity	Problems in identifying hydrocarbons that produce similar ions Highly manual workflows for sample preparation can benefit from automation The high complexity of the instrumentation’s operation and maintenance when looking at a limited number of analytes	Proteins Metabolites Lipids
GC-MS [147,150]	High-efficiency separations Suitable for nonpolar, volatile, and small molecules High sensitivity High throughput	Limited mass range Limited to thermally stable and volatile compounds Destructive analysis Not suitable for compounds heavier than 1000 Da Time-consuming for sample preparation	Metabolites Lipids
NMR [150,151]	Very high reproducibility High throughput Non-destructive Sample recovery Rapid	Highly skilled operators Low sensitivity Cost is higher than GC-MS and LC-MS Difficult to quantify the noise.	Metabolites
1DGE [68,152,153,154]	Simple workflow Rapid Cost-effective	Limited reproducibility Unsuitable for low-abundance proteins Hydrophobic proteins’ insolubility	Proteins
2DGE [68,152,153,154]	High resolution High throughput Cost-effective	Gel-to-gel variation Lack of sensitivity Poor dynamic range Time-consuming Highly skilled operators Not automated approach	Proteins
2D-DIGE [153,155,156]	Wide dynamic range detection Fewer number of gels Straightforward matching between gels Higher sensitivity and reproducibility over 2DGE	Highly skilled operators Time-consuming Lower throughput Not suitable for extremely acidic, basic, or hydrophobic proteins	Proteins
Immunoassay techniques (ELISA, Western Blot) [152,157,158]	High sensitivity and specificity when looking at a limited number of analytes Cost-effective Simple workflows Highly reproducible Suitable for validation	Resource-intensive efforts Time-consuming Not recognition of posttranslational protein variants Limited multiplexing options Relatively high sample volume Cross-reactivity Stability of reagents affects outcome Limited number of analytes in each analysis	Proteins

## Data Availability

All data generated or analysed during this study are included either as a supplementary file or are publicly available and properly referenced in the manuscript.

## Supplementary Materials

The following supporting information can be downloaded at: https://www.mdpi.com/article/10.3390/cancers15102780/s1, Supplementary Table S1: The titles of metabolomic, lipidomic, proteomic, and multi-omics investigations carried out on various biofluid samples of breast cancer between January 2001 and April 2023; Supplementary Table S2: The details of the approaches undertaken in relation to the discovery of non-nucleotide biomarkers in breast cancer investigations (selected studies).
