# Supplementary material for: Biomarker Reproducibility Challenge: A Review of Non-Nucleotide Biomarker Discovery Protocols from Body Fluids in Breast Cancer Diagnosis
Source: Cancers (Basel). 2023 May 16;15(10):2780. doi: 10.3390/cancers15102780 (PMC10216598; doi:10.3390/cancers15102780)
Supplement: Supplementary file 1 [file cancers-15-02780-s001.zip › Supplementary Table S1.pdf]

Proteomics studies using liquid biopsy on breast cancer			
**Sample Type**	**Year of Publication**	**Biomarker Type**	**Title**
Urine	2016	Proteomics	Urinary proteome alterations in HER2 enriched breast cancer revealed by multipronged quantitative proteomics.
Urine	2015	Proteomics	Proteomic Analysis of Urine to Identify Breast Cancer Biomarker Candidates Using a Label-Free LC-MS/MS Approach.
Urine	2009	Proteomics	Autoantibody to tumor antigen, alpha 2-HS glycoprotein: a novel biomarker of breast cancer screening and diagnosis
Tear	2012	Proteomics	Comparison of tear protein levels in breast cancer patients and healthy controls using a de novo proteomic approach.
Tear	2022	Proteomics	Using tears as a non-invasive source for early detection of breast cancer
Tear	2009	Proteomics	Surface-enhanced Laser Desorption/Ionisation Time-of-flight Mass Spectrometry to Detect Breast Cancer Markers in Tears and Serum.
Tear	2009	Proteomics	Diagnosis of breast cancer by tear proteomic pattern.
Tear	2001	Proteomics	Lacryglobin in human tears, a potential marker for cancer
Serum	2023	proteomics	Cancer Serum Atlas-Supported Precise Pan-Targeted Proteomics Enable Multicancer Detection
Serum	2023	Proteomics	Potential Early Markers for Breast Cancer: A Proteomic Approach Comparing Saliva and Serum Samples in a Pilot Study
Serum	2022	Proteomics	Apolipoprotein C1 (APOC1), A Candidate Diagnostic Serum Biomarker for Breast Cancer Identified by Serum Proteomics Study.
Serum	2022	Proteomics	Exploration of quantitative site-specific serum O-glycoproteomics with isobaric labeling for the discovery of putative O-glycoprotein biomarkers
Serum	2020	Proteomics	Shotgun proteomics coupled to nanoparticle-based biomarker enrichment reveals a novel panel of extracellular matrix proteins as candidate serum protein biomarkers for early-stage breast cancer detection.
Serum	2020	Proteomics	Quantifying Serum Derived Differential Expressed and Low Molecular Weight Protein in Breast Cancer Patients.
Serum	2020	Proteomics	Proteomic investigation on bio-corona of Au, Ag and Fe nanoparticles for the discovery of triple negative breast cancer serum protein biomarkers.
Serum	2019	Proteomics	A Novel Strategy to Investigate Tissue-Secreted Tumor Microenvironmental Proteins in Serum toward Development of Breast Cancer Early Diagnosis Biomarker Signature.
Serum	2018	Proteomics	An efficient biomarker panel for diagnosis of breast cancer using surface-enhanced laser desorption ionization time-of-flight mass spectrometry.
Serum	2017	Proteomics	Identification of potential serum biomarkers for breast cancer using a functional proteomics technology.
Serum	2017	Proteomics	Detection and identification of serum protein peak at 6648 m/z as a novel indicator in breast cancer based on mass spectrometry.
Serum	2016	Proteomics	Identification of novel serum peptides biomarkers for female breast cancer patients in Western China.
Serum	2016	Proteomics	Diagnostic and prognostic significance of serum apolipoprotein C-I in triple-negative breast cancer based on mass spectrometry.
Serum	2015	Proteomics	Alpha 2HS-glycoprotein, a tumor-associated antigen (TAA) detected in Mexican patients with early-stage breast cancer.
Serum	2015	Proteomics	Proteomic Profiling of Invasive Ductal Carcinoma (IDC) using Magnetic Beads-based Serum Fractionation and MALDI-TOF MS.
Serum	2014	Proteomics	Novel serum protein biomarker panel revealed by mass spectrometry and its prognostic value in breast cancer.
Serum	2014	Proteomics	Analysis of the differences of serum protein mass spectrometry in patients with triple negative breast cancer and non-triple negative breast cancer.
Serum	2014	Proteomics	Dermcidin expression is associated with disease progression and survival among breast cancer patients
Serum	2014	Proteomics	Association of N-glycosylation with breast carcinoma and systemic features using high-resolution quantitative UPLC
Serum	2014	Proteomics	Serum and saliva protein levels in females with breast cancer.
Serum	2013	Proteomics	Proteomic profile in familial breast cancer patients.
Serum	2013	Proteomics	Proteomic profiling of triple-negative breast carcinomas in combination with a three-tier orthogonal technology approach identifies Mage-A4 as potential therapeutic target in estrogen receptor negative breast cancer.
Serum	2012	Proteomics	Serum protein levels following surgery in breast cancer patients: a protein microarray approach.
Serum	2012	Proteomics	Serum peptidome patterns of breast cancer based on magnetic bead separation and mass spectrometry analysis.
Serum	2012	Proteomics	Body mass index and serum proteomic profile in breast cancer and healthy women: a prospective study.
Serum	2012	Proteomics	Alpha 1-antitrypsin: a novel tumor-associated antigen identified in patients with early-stage breast cancer.
Serum	2012	Proteomics	Potential biomarkers in the sera of breast cancer patients from bahawalpur, pakistan.
Serum	2012	Proteomics	[Identification of a novel serum marker for early breast cancer detection by proteomic approach]
Serum	2011	Proteomics	A proteomics platform combining depletion, multi-lectin affinity chromatography (M-LAC), and isoelectric focusing to study the breast cancer proteome
Serum	2011	Proteomics	Serum proteome profiling of primary breast cancer indicates a specific biomarker profile.
Serum	2011	Proteomics	Searching for early breast cancer biomarkers by serum protein profiling of pre-diagnostic serum; a nested case-control study.
Serum	2011	Proteomics	Antibody microarray analysis of the serum proteome in primary breast cancer patients
Serum	2011	Proteomics	Primary study of lymph node metastasis-related serum biomarkers in breast cancer.
Serum	2011	Proteomics	Identification of vitronectin as a novel serum marker for early breast cancer detection using a new proteomic approach.
Serum	2011	Proteomics	Differential protein expression in primary breast cancer and matched axillary node metastasis
Serum	2011	Proteomics	Protein microarray signature of autoantibody biomarkers for the early detection of breast cancer.
Serum	2010	Proteomics	Optimizing of MALDI-ToF-based low-molecular-weight serum proteome pattern analysis in detection of breast cancer patients; the effect of albumin removal on classification performance.
Serum	2010	Proteomics	Mass spectrometry-based analysis of therapy-related changes in serum proteome patterns of patients with early-stage breast cancer.
Serum	2010	Proteomics	Detection and identification of potential biomarkers of breast cancer
Serum	2009	Proteomics	Influence of sample storage duration on serum protein profiles assessed by surface-enhanced laser desorption/ionisation time-of-flight mass spectrometry (SELDI-TOF MS).
Serum	2009	Proteomics	Mass spectrometry-based serum proteome pattern analysis in molecular diagnostics of early stage breast cancer.
Serum	2009	Proteomics	Proteomic analysis of archival breast cancer serum.
Serum	2009	Proteomics	Serum proteomic biomarker discovery reflective of stage and obesity in breast cancer patients

Sample Type	Year of Publication	Biomarker Type	Title
Serum	2009	Proteomics	The multiplex bead array approach to identifying serum biomarkers associated with breast cancer.
Serum	2009	Proteomics	Detection of breast cancer by surface-enhanced laser desorption/ionization time-of-flight mass spectrometry tissue and serum protein profiling.
Serum	2009	Proteomics	Proteomics-based identification of alpha1-antitrypsin and haptoglobin precursors as novel serum markers in infiltrating ductal breast carcinomas.
Serum	2009	Proteomics	Mass spectrometry-based serum proteome pattern analysis in molecular diagnostics of early stage breast cancer.
Serum	2009	Proteomics	Surface-enhanced Laser Desorption/Ionisation Time-of-flight Mass Spectrometry to Detect Breast Cancer Markers in Tears and Serum.
Serum	2009	Proteomics	Identification of a new panel of serum autoantibodies associated with the presence of in situ carcinoma of the breast in younger women.
Serum	2008	Proteomics	Case-control breast cancer study of MALDI-TOF proteomic mass spectrometry data on serum samples.
Serum	2008	Proteomics	Serum proteome profiling of metastatic breast cancer using recombinant antibody microarrays.
Serum	2008	Proteomics	Cytokine comparisons between women with breast cancer and women with a negative breast biopsy
Serum	2008	Proteomics	Combined experimental and statistical strategy for mass spectrometry based serum protein profiling for diagnosis of breast cancer: a case-control study.
Serum	2007	Proteomics	Serum proteomic analysis identifies a highly sensitive and specific discriminatory pattern in stage 1 breast cancer.
Serum	2006	Proteomics	MALDI-TOF serum protein profiling for the detection of breast cancer.
Serum	2006	Proteomics	Serum biomarkers for detection of breast cancers: A prospective study.
Serum	2005	Proteomics	Analytical and preanalytical biases in serum proteomic pattern analysis for breast cancer diagnosis.
Serum	2005	Proteomics	Genomics, Proteomics and Cancer: Specific Ribosomal, Mitochondrial, and Tumor Reactive Proteins Can Be Used as Biomarkers for Early Detection of Breast Cancer in Serum
Serum	2005	Proteomics	SELDI-TOF-MS: the proteomics and bioinformatics approaches in the diagnosis of breast cancer.
Serum	2005	Proteomics	Analytical and Preanalytical Biases in Serum Proteomic Pattern Analysis for Breast Cancer Diagnosis
Serum	2003	Proteomics	Use of serological proteomic methods to find biomarkers associated with breast cancer.
Serum	2002	Proteomics	Proteomics and bioinformatics approaches for identification of serum biomarkers to detect breast cancer.
Saliva	2023	Proteomics	Potential Early Markers for Breast Cancer: A Proteomic Approach Comparing Saliva and Serum Samples in a Pilot Study
Saliva	2022	Proteomics	Targeted proteomics using parallel reaction monitoring confirms salivary proteins indicative of metastatic triple-negative breast cancer
Saliva	2019	Proteomics	In search of the altering salivary proteome in metastatic breast and ovarian cancers
Saliva	2015	Proteomics	Surface-enhanced Raman spectroscopy of saliva proteins for the noninvasive differentiation of benign and malignant breast tumors.
Saliva	2014	Proteomics	Serum and saliva protein levels in females with breast cancer.
Saliva	2012	Proteomics	Salivary Protein Profiles among HER2/neu-Receptor-Positive and -Negative Breast Cancer Patients: Support for Using Salivary Protein Profiles for Modeling Breast Cancer Progression.
Saliva	2010	Proteomics	Discovery and preclinical validation of salivary transcriptomic and proteomic biomarkers for the non-invasive detection of breast cancer.
Saliva	2009	Proteomics	A Comparison of the Proteomic Expression in Pooled Saliva Specimens from Individuals Diagnosed with Ductal Carcinoma of the Breast with and without Lymph Node Involvement.
Saliva	2008	Proteomics	Altered biochemical parameters in the saliva of patients with breast cancer
Saliva	2008	Proteomics	Breast cancer related proteins are present in saliva and are modulated secondary to ductal carcinoma in situ of the breast.
Saliva	2006	Proteomics	The use of surface-enhanced laser desorption/ionization time-of-flight mass spectrometry to detect putative breast cancer markers in saliva: a feasibility study
Plasma	2021	Proteomics	Identification of blood protein biomarkers for breast cancer staging by integrative transcriptome and proteome analyses.
Plasma	2022	Proteomics	Integrative analysis of plasma metabolomics and proteomics reveals the metabolic landscape of breast cancer
Plasma	2020	Proteomics	Identification of novel alternative splicing biomarkers for breast cancer with LC/MS/MS and RNA-Seq.
Plasma	2022	Proteomics	Internal Standard Triggered-Parallel Reaction Monitoring Mass Spectrometry Enables Multiplexed Quantification of Candidate Biomarkers in Plasma
Plasma	2020	Proteomics	Proteomic analysis of circulating extracellular vesicles identifies potential markers of breast cancer progression, recurrence, and response.
Plasma	2019	Proteomics	The plasma peptides of breast versus ovarian cancer.
Plasma	2019	Proteomics	Plasma Peptidome Pattern of Breast Cancer Using Magnetic Beads-Based Plasma Fractionation and MALDI-TOF MS: A Case Control Study in Egypt.
Plasma	2019	Proteomics	A plasma protein derived TGFβ signature is a prognostic indicator in triple negative breast cancer.
Plasma	2018	Proteomics	Affinity proteomic profiling of plasma for proteins associated to area-based mammographic breast density.
Plasma	2018	Proteomics	Label-Free Quantitative Proteomic Screening of Candidate Plasma Biomarkers for the Prognosis of Breast Cancer with Different Lymph Node Statuses.
Plasma	2017	Proteomics	Phosphoproteins in extracellular vesicles as candidate markers for breast cancer.
Plasma	2017	Proteomics	Identifying potential markers in Breast Cancer subtypes using plasma label-free proteomics
Plasma	2017	Proteomics	Label-Free Proteome Analysis of Plasma from Patients with Breast Cancer: Stage-Specific Protein Expression
Plasma	2016	Proteomics	Quantitative proteomics revealed novel proteins associated with molecular subtypes of breast cancer.
Plasma	2016	Proteomics	Identification of Developmental Endothelial Locus-1 on Circulating Extracellular Vesicles as a Novel Biomarker for Early Breast Cancer Detection.
Plasma	2015	Proteomics	Plasma Proteomic Profiling in Hereditary Breast Cancer Reveals a BRCA1-Specific Signature: Diagnostic and Functional Implications
Plasma	2015	Proteomics	Development and Validation of a Novel Plasma Protein Signature for Breast Cancer Diagnosis by Using Multiple Reaction Monitoring-based Mass Spectrometry
Plasma	2015	Proteomics	Targeted mass spectrometry analysis of the proteins IGF1, IGF2, IBP2, IBP3 and A2GL by blood protein precipitation
Plasma	2014	Proteomics	Phosphoprotein secretome of tumor cells as a source of candidates for breast cancer biomarkers in plasma
Plasma	2014	Proteomics	Hepcidin and ferritin blood level as noninvasive tools for predicting breast cancer.
Plasma	2013	Proteomics	Breast cancer subtyping from plasma proteins
Plasma	2013	Proteomics	A neural network approach to multi-biomarker panel discovery by high-throughput plasma proteomics profiling of breast cancer.

Sample Type	Year of Publication	Biomarker Type	Title
Plasma	2013	Proteomics	Novel alternative splicing isoform biomarkers identification from high-throughput plasma proteomics profiling of breast cancer
Plasma	2013	Proteomics	A mass spectrometry-based plasma protein panel targeting the tumor microenvironment in patients with breast cancer
Plasma	2013	Proteomics	A Validation Study of a Multiple Reaction Monitoring-Based Proteomic Assay to Diagnose Breast Cancer
Plasma	2012	Proteomics	Comparative profiling of plasma proteome from breast cancer patients reveals thrombospondin-1 and BRWD3 as serological biomarkers.
Plasma	2012	Proteomics	Surface plasmon resonance imaging in arrays coupled with mass spectrometry (SUPRA-MS): proof of concept of on-chip characterization of a potential breast cancer marker in human plasma
Plasma	2011	Proteomics	A large, consistent plasma proteomics data set from prospectively collected breast cancer patient and healthy volunteer samples
Plasma	2011	Proteomics	Discovery and validation of breast cancer early detection biomarkers in preclinical samples
Plasma	2010	Proteomics	Differential profiling of breast cancer plasma proteome by isotope-coded affinity tagging method reveals biotinidase as a breast cancer biomarker.
Plasma	2010	Proteomics	Detection of elevated plasma levels of epidermal growth factor receptor before breast cancer diagnosis among hormone therapy users
Plasma	2010	Proteomics	Blood peptidome-degradome profile of breast cancer.
Plasma	2009	Proteomics	Intrinsic subtype-associated changes in the plasma proteome in breast cancer
NAF	2021	Proteomics	High-Throughput Proteomic Profiling of Nipple Aspirate Fluid from Breast Cancer Patients Compared with Non-Cancer Controls: A Step Closer to Clinical Feasibility.
NAF	2017	Proteomics	Nipple aspirate fluid-A liquid biopsy for diagnosing breast health
NAF	2016	Proteomics	Protein Biomarkers for Breast Cancer Risk Are Specifically Correlated with Local Steroid Hormones in Nipple Aspirate Fluid.
NAF	2015	Proteomics	Repeated nipple fluid aspiration: compliance and feasibility results from a prospective multicenter study.
NAF	2015	Proteomics	Protein identification from dried nipple aspirate fluid on Guthrie cards using mass spectrometry.
NAF	2014	Proteomics	Potential correlation between tumor aggressiveness and protein expression patterns of nipple aspirate fluid (NAF) revealed by gel-based proteomic analysis.
NAF	2013	Proteomics	Resolving breast cancer heterogeneity by searching reliable protein cancer biomarkers in the breast fluid secretome.
NAF	2012	Proteomics	Proteins and carbohydrates in nipple aspirate fluid predict the presence of atypia and cancer in women requiring diagnostic breast biopsy.
NAF	2010	Proteomics	Nipple aspirate fluid proteome of healthy females and patients with breast cancer.
NAF	2009	Proteomics	Identification of a beta-casein-like peptide in breast nipple aspirate fluid that is associated with breast cancer.
NAF	2007	Proteomics	A comparative proteinomic analysis of nipple aspiration fluid from healthy women and women with breast cancer.
NAF	2007	Proteomics	Proteomics of nipple aspirate fluid, breast cyst fluid, milk, and colostrum.
NAF	2007	Proteomics	Biologic markers of breast cancer in nipple aspirate fluid and nipple discharge are associated with clinical findings.
NAF	2007	Proteomics	Detection of breast cancer biomarkers in nipple aspirate fluid by SELDI-TOF and their identification by combined liquid chromatography-tandem mass spectrometry.
NAF	2006	Proteomics	Proteomic analysis of nipple aspirate fluid from women with early-stage breast cancer using isotope-coded affinity tags and tandem mass spectrometry reveals differential expression of vitamin D binding protein.
NAF	2005	Proteomics	Identification of biomarkers for breast cancer in nipple aspiration and ductal lavage fluid
NAF	2005	Proteomics	Significant differences in nipple aspirate fluid protein expression between healthy women and those with breast cancer demonstrated by time-of-flight mass spectrometry.
NAF	2005	Proteomics	Proteomic analysis of nipple aspirate fluid using SELDI-TOF-MS.
NAF	2004	Proteomics	Proteomic analysis to identify breast cancer biomarkers in nipple aspirate fluid.
NAF	2004	Proteomics	Association between ductal fluid proteomic expression profiles and the presence of lymph node metastases in women with breast cancer.
NAF	2003	Proteomics	Proteomic characterization of nipple aspirate fluid: identification of potential biomarkers of breast cancer.
NAF	2003	Proteomics	Proteomic characterization of nipple aspirate fluid: identification of potential biomarkers of breast cancer.
NAF	2002	Proteomics	Proteomic analysis of nipple aspirate fluid to detect biologic markers of breast cancer.
NAF	2002	Proteomics	Identification of distinct protein expression patterns in bilateral matched pair breast ductal fluid specimens from women with unilateral invasive breast carcinoma. High-throughput biomarker discovery.
NAF	2001	Proteomics	Proteomic patterns of nipple aspirate fluids obtained by SELDI-TOF: potential for new biomarkers to aid in the diagnosis of breast cancer.
DLF	2005	Proteomics	Identification of biomarkers for breast cancer in nipple aspiration and ductal lavage fluid
DLF	2005	Proteomics	Cytologic findings and protein expression profiles associated with ductal carcinoma of the breast in ductal lavage specimens using surface-enhanced laser desorption and ionization-time of flight mass spectrometry.

Metabolomics studies using liquid biopsy on breast cancer			
**Sample Type**	**Year of Publication**	**Biomarker Type**	**Title**
Urine	2022	Metabolomics	Blood and urine biomarkers in invasive ductal breast cancer: Mass spectrometry applied to identify metabolic alterations
Urine	2012	Metabolomics	Solid phase microextraction, mass spectrometry and metabolomic approaches for detection of potential urinary cancer biomarkers-A powerful strategy for breast cancer diagnosis
Urine	2019	Metabolomics	Implementing a central composite design for the optimization of solid phase microextraction to establish the urinary volatomic expression: a first approach for breast cancer
Urine	2021	Metabolomics	An integrative approach based on GC-qMS and NMR metabolomics data as a comprehensive strategy to search potential breast cancer biomarkers
Urine	2018	Metabolomics	Exploring the potential of needle trap microextraction combined with chromatographic and statistical data to discriminate different types of cancer based on urinary volatomic biosignature
Urine	2018	Metabolomics	A non-invasive approach to explore the discriminatory potential of the urinary volatilome of invasive ductal carcinoma of the breast†
Urine	2022	Metabolomics	Breast cancer detection by analyzing the volatile organic compound (VOC) signature in human urine
Urine	2019	Metabolomics	Urinary Metabolites as Biomarkers for Diagnosis of Breast Cancer: A Preliminary Study
Urine	2010	Metabolomics	Multivariate classification of urine metabolome profiles for breast cancer diagnosis.
Urine	2014	Metabolomics	Urinary prostaglandin E2 metabolite and breast cancer risk.
Urine	2005	Metabolomics	Urinary hydroxyestrogens and breast cancer risk among postmenopausal women: a prospective study
Urine	2010	Metabolomics	Comparison of estrogens and estrogen metabolites in human breast tissue and urine.
Urine	2010	Metabolomics	Evaluating the utility of N1,N12-diacetylspermine and N1,N8-diacetylspermidine in urine as tumor markers for breast and colorectal cancers
Urine	2010	Metabolomics	Urine metabolite analysis offers potential early diagnosis of ovarian and breast cancers
Urine	2018	Metabolomics	Early non-invasive detection of breast cancer using exhaled breath and urine analysis
Urine	2018	Metabolomics	Urinary metabolite and lipid alterations in Colombian Hispanic women with breast cancer: A pilot study.
Urine	2019	Metabolomics	Untargeted Urinary 1H NMR-Based Metabolomic Pattern as a Potential Platform in Breast Cancer Detection.
Urine	2009	Metabolomics	Mass spectrometry based metabolomic approaches in urinary biomarker study of women's cancers.
NAF	2014	Metabolomics	Metabolomic Characterization of Nipple Aspirate Fluid by 1H NMR Spectroscopy and GC-MS
DLF	2013	Metabolomics	Metabolomic profiling reveals significant differences between the ductal fluid from cancerous compared to unaffected breasts
DLF	2016	Metabolomics	Metabolomic profiling of breast tumors using ductal fluid
DLF	2010	Metabolomics	Breast ductal lavage for assessment of breast cancer biomarkers
Serum	2021	Metabolomics	Serum N-glycan profiles differ for various breast cancer subtypes
Serum	2023	Metabolomics	A metabolome-wide case-control study of african american breast cancer patients
serum	2023	Metabolomics	Association of altered metabolic profiles and long non-coding RNAs expression with disease severity in breast cancer patients: analysis by 1H NMR spectroscopy and RT-q-PCR
Serum	2023	Metabolomics	Association of serum metabolome profile with the risk of breast cancer in participants of the HUNT2 study
Serum	2023	Metabolomics	Risk assessment of disease recurrence in early breast cancer: A serum metabolomic study focused on elderly patients
Serum	2022	Metabolomics	A non-invasive method for concurrent detection of early-stage women-specific cancers
Serum	2022	Metabolomics	Screening and diagnosis of triple negative breast cancer based on rapid metabolic fingerprinting by conductive polymer spray ionization mass spectrometry and machine learning
Serum	2022	Metabolomics	Two effective models based on comprehensive lipidomics and metabolomics can distinguish BC versus HCs, and TNBC versus non-TNBC
Serum	2022	Metabolomics	Metabolomic study of serum in patients with invasive ductal breast carcinoma with LC-MS/MS approach
Serum	2022	Metabolomics	Blood and urine biomarkers in invasive ductal breast cancer: Mass spectrometry applied to identify metabolic alterations
Serum	2022	Metabolomics	Untargeted metabolomics based on nuclear magnetic resonance spectroscopy and multivariate classification techniques for identifying metabolites associated with breast cancer patients
Serum	2017	Metabolomics	Serum Metabolomic Profiles for Breast Cancer Diagnosis, Grading and Staging by Gas Chromatography-Mass Spectrometry
Serum	2007	Metabolomics	A serum glycomics approach to breast cancer biomarkers.
Serum	2020	Metabolomics	A novel serum metabolome score for breast cancer diagnosis.
Serum	2021	Metabolomics	Relationship between the n-3 index, serum metabolites and breast cancer risk.
Serum	2021	Metabolomics	Altered Serum Metabolic Profile Assessed by Advanced 1H-NMR in Breast Cancer Patients.
Serum	2019	Metabolomics	Assessing Treatment Response and Prognosis by Serum and Tissue Metabolomics in Breast Cancer Patients.
Serum	2018	Metabolomics	A Metabolomics Analysis of Body Mass Index and Postmenopausal Breast Cancer Risk.
Serum	2016	Metabolomics	Serum metabolomics analysis reveals changes in signaling lipids in breast cancer patients.
Serum	2017	Metabolomics	Serum Metabolomic Profiles for Breast Cancer Diagnosis, Grading and Staging by Gas Chromatography-Mass Spectrometry.
Serum	2017	Metabolomics	1H NMR Metabolomics Reveals Association of High Expression of Inositol 1, 4, 5 Trisphosphate Receptor and Metabolites in Breast Cancer Patients.
Serum	2017	Metabolomics	Serum Metabolomic Profiles Identify ER-Positive Early Breast Cancer Patients at Increased Risk of Disease Recurrence in a Multicenter Population.
Serum	2021	Metabolomics	Identification of Novel Diagnostic Biomarkers in Breast Cancer Using Targeted Metabolomic Profiling.
Serum	2012	Metabolomics	Exploration of serum metabolomic profiles and outcomes in women with metastatic breast cancer: a pilot study.
Serum	2015	Metabolomics	Serum metabolomic profiles evaluated after surgery may identify patients with oestrogen receptor negative early breast cancer at increased risk of disease recurrence. Results from a retrospective study.
Serum	2018	Metabolomics	Taurine, glutamic acid and ethylmalonic acid as important metabolites for detecting human breast cancer based on the targeted metabolomics

Sample Type	Year of Publication	Biomarker Type	Title
Serum	2014	Metabolomics	A serum nuclear magnetic resonance-based metabolomic signature of advanced metastatic human breast cancer.
Serum	2021	Metabolomics	Prediagnostic circulating metabolites in female breast cancer cases with low and high mammographic breast density.
Serum	2011	Metabolomics	Identification of a serum-detectable metabolomic fingerprint potentially correlated with the presence of micrometastatic disease in early breast cancer patients at varying risks of disease relapse by traditional prognostic methods
Serum	2018	Metabolomics	Analysis of metabolites and metabolic pathways in breast cancer in a Korean prospective cohort: the Korean Cancer Prevention Study-II
Serum	2022	Metabolomics	Circulating metabolites serve as diagnostic biomarkers for HER2-positive breast cancer and have predictive value for trastuzumab therapy outcomes.
Serum	2017	Metabolomics	Does the 1H-NMR plasma metabolome reflect the host-tumor interactions in human breast cancer?
Serum	2022	Metabolomics	Evaluation of Untargeted Metabolomic Strategy for the Discovery of Biomarker of Breast Cancer.
Plasma	2023	Metabolomics	A prospective case-cohort analysis of plasma metabolites and breast cancer risk
Plasma	2022	Metabolomics	A Metabolomics Analysis of Circulating Carotenoids and Breast Cancer Risk
Plasma	2021	Metabolomics	Early Breast Cancer Detection Using Untargeted and Targeted Metabolomics.
Plasma	2021	Metabolomics	Metabolomic analysis of plasma from breast tumour patients. A pilot study
Plasma	2022	Metabolomics	Integrative analysis of plasma metabolomics and proteomics reveals the metabolic landscape of breast cancer
Plasma	2018	Metabolomics	Metabolic fingerprinting in breast cancer stages through 1H NMR spectroscopy-based metabolomic analysis of plasma.
Plasma	2022	Metabolomics	Metabolomic Analysis of Plasma from Breast Cancer Patients Using Ultra-High-Performance Liquid Chromatography Coupled with Mass Spectrometry: An Untargeted Study.
Plasma	2018	Metabolomics	Breast cancer detection using targeted plasma metabolomics.
Plasma	2017	Metabolomics	A plasma metabolomic signature discloses human breast cancer.
Plasma	2019	Metabolomics	Plasma metabolites as possible biomarkers for diagnosis of breast cancer.
Plasma	2015	Metabolomics	Forecasting individual breast cancer risk using plasma metabolomics and biocontours.
Plasma	2022	Metabolomics	Plasma Metabolomics and Breast Cancer Risk over 20 Years of Follow-up among Postmenopausal Women in the Nurses' Health Study.
Plasma	2019	Metabolomics	A plasma metabolite panel as biomarkers for early primary breast cancer detection.
Plasma	2019	Metabolomics	Plasma Metabolomic Signatures Associated with Long-term Breast Cancer Risk in the SU.VI.MAX Prospective Cohort.
Plasma	2021	Metabolomics	Investigation of circulating metabolites associated with breast cancer risk by untargeted metabolomics: a case-control study nested within the French E3N cohort.
Plasma	2018	Metabolomics	NMR metabolomic signatures reveal predictive plasma metabolites associated with long-term risk of developing breast cancer.
Plasma	2016	Metabolomics	Human plasma metabolomics for identifying differential metabolites and predicting molecular subtypes of breast cancer.
Plasma	2018	Metabolomics	Multiplatform plasma metabolic and lipid fingerprinting of breast cancer: A pilot control-case study in Colombian Hispanic women.
Plasma	2019	Metabolomics	Prospective analysis of circulating metabolites and breast cancer in EPIC.
Plasma	2022	Metabolomics	Diagnostic Value of 1 H NMR-Based Metabolomics in Acute Lymphoblastic Leukemia, Acute Myeloid Leukemia, and Breast Cancer
Plasma	2015	Metabolomics	Lowered circulating aspartate is a metabolic feature of human breast cancer
Plasma	2010	Metabolomics	Early detection of recurrent breast cancer using metabolite profiling.
Plasma	2009	Metabolomics	[Analysis of serum metabonome of patients with breast cancer by gas chromatography-mass spectrometry].
Saliva	2022	Metabolomics	Metabolic Features of Saliva in Breast Cancer Patients.
Saliva	2014	Metabolomics	Establishment of the Saliva Volatomic Profile as an Exploratory and Non-invasive Strategy to Find Potential Breast Cancer Biomarkers
Saliva	2019	Metabolomics	Salivary metabolomics with alternative decision tree-based machine learning methods for breast cancer discrimination
Saliva	2016	Metabolomics	Untargeted saliva metabonomics study of breast cancer based on ultra performance liquid chromatography coupled to mass spectrometry with HILIC and RPLC separations.
Saliva	2020	Metabolomics	Using an Untargeted Metabolomics Approach to Identify Salivary Metabolites in Women with Breast Cancer
Saliva	2018	Metabolomics	Screening of salivary volatiles for putative breast cancer discrimination: an exploratory study involving geographically distant populations
Saliva	2022	Metabolomics	«Salivaomics» of Different Molecular Biological Subtypes of Breast Cancer
Tear	2020	Metabolomics	Label-Free Surface-Enhanced Raman Spectroscopy Biosensor for On-Site Breast Cancer Detection Using Human Tears

Lipidomic studies using liquid biopsy on breast cancer			
**Sample Type**	**Year of Publication**	**Biomarker Type**	**Title**
Serum	2022	Lipidomics	Two effective models based on comprehensive lipidomics and metabolomics can distinguish BC versus HCs, and TNBC versus non-TNBC
Serum	2010	Lipidomics	Metabolic profile, physical activity, and mortality in breast cancer patients
Serum	2021	Lipidomics	Altered Serum Metabolic Profile Assessed by Advanced 1H-NMR in Breast Cancer Patients
Serum	2014	Lipidomics	Decreased serum levels of free fatty acids are associated with breast cancer
Serum	2009	Lipidomics	Elevated levels of hydroxylated phosphocholine lipids in the blood serum of breast cancer patients
Serum	2016	Lipidomics	Serum metabolomics analysis reveals changes in signaling lipids in breast cancer patients.
Plasma	2020	Lipidomics	Triple Negative Breast Cancer Detection Using LC-MS/MS Lipidomic Profiling.
Plasma	2022	Lipidomics	Multi-Omics Approach Points to the Importance of Oxylipins Metabolism in Early-Stage Breast Cancer
Plasma	2021	Lipidomics	Plasma lipidomic profiles of kidney, breast and prostate cancer patients differ from healthy controls.
Plasma	2020	Lipidomics	Multiplatform Investigation of Plasma and Tissue Lipid Signatures of Breast Cancer Using Mass Spectrometry Tools
Plasma	2018	Lipidomics	Multiplatform plasma metabolic and lipid fingerprinting of breast cancer: A pilot control-case study in Colombian Hispanic women.
Plasma	2013	Lipidomics	Mass spectrometry-based quantitative metabolomics revealed a distinct lipid profile in breast cancer patients.
Plasma	2018	Lipidomics	Metabolomic profiles in breast cancer:a pilot case-control study in the breast cancer family registry.
Plasma	2022	Lipidomics	Liquid biopsy markers for early diagnosis of brain metastasis patients with breast cancer by metabolomics.
Plasma	2017	Lipidomics	Potential plasma lipid biomarkers in early-stage breast cancer.
Plasma	2021	Lipidomics	1 H-NMR Plasma Lipoproteins Profile Analysis Reveals Lipid Metabolism Alterations in HER2-Positive Breast Cancer Patients
Plasma	2016	Lipidomics	Plasma lipidomics profiling identified lipid biomarkers in distinguishing early-stage breast cancer from benign lesions.
Plasma	2022	Lipidomics	Multi-Omics Approach Points to the Importance of Oxylipins Metabolism in Early-Stage Breast Cancer
Plasma	2020	Lipidomics	Rapid profiling method for the analysis of lipids in human plasma using ion mobility enabled-reversed phase-ultra high performance liquid chromatography/mass spectrometry
Plasma	2007	Lipidomics	Liquid chromatography mass spectrometry for quantifying plasma lysophospholipids: potential biomarkers for cancer diagnosis
Plasma	2015	Lipidomics	Comprehensive lipid profiling of plasma in patients with benign breast tumor and breast cancer reveals novel biomarkers.
Urine	2018	Lipidomics	Urinary metabolite and lipid alterations in Colombian Hispanic women with breast cancer: A pilot study.
Urine	2010	Lipidomics	Quantitative analysis of urinary phospholipids found in patients with breast cancer by nanoflow liquid chromatography-tandem mass spectrometry: II. Negative ion mode analysis of four phospholipid classes
Urine	2009	Lipidomics	Quantitative analysis of phosphatidylcholines and phosphatidylethanolamines in urine of patients with breast cancer by nanoflow liquid chromatography/tandem mass spectrometry
Saliva	2021	Lipidomics	Analysis of Saliva Lipids in Breast and Prostate Cancer by IR Spectroscopy.

Proteomics, Metabolomics, and Lipidomics studies using Evs, MVs, and Exosomes on breast cancer			
**Sample Type**	**Year of Publication**	**Omic**	**Title**
Plasma-Evs	2020	Proteomics	Proteomic analysis of circulating extracellular vesicles identifies potential markers of breast cancer progression, recurrence, and response
Plasma-Evs	2017	Proteomics	Phosphoproteins in extracellular vesicles as candidate markers for breast cancer
Plasma-Evs	2018	Proteomics	Profiling plasma extracellular vesicle by pluronic block-copolymer based enrichment method unveils features associated with breast cancer aggression, metastasis and invasion
Plasma-Evs	2020	Proteomics	Extracellular vesicles from young women's breast cancer patients drive increased invasion of non-malignant cells via the Focal Adhesion Kinase pathway: a proteomic approach
Plasma-Evs	2016	Proteomics	Identification of Developmental Endothelial Locus-1 on Circulating Extracellular Vesicles as a Novel Biomarker for Early Breast Cancer Detection
Plasma-Evs	2021	Proteomics	Early Detection and Investigation of Extracellular Vesicles Biomarkers in Breast Cancer
Plasma-Evs	2021	Lipidomics	Lipidomic analysis of extracellular vesicles and its potential for the identification of body fluid-based biomarkers for breast cancer diagnosis
Plasma-MV	2021	Metabolomics	Metabolomic Profiling of Blood-Derived Microvesicles in Breast Cancer Patients
Plasma-Exosome	2021	Proteomics	Deep proteomic analysis of plasma exosomes in patients with advanced, hormone receptor-positive breast cancer treated with palbociclib and tamoxifen.
Plasma-Exosome	2022	Metabolomics	Exosomal Metabolic Signatures Are Associated with Differential Response to Neoadjuvant Chemotherapy in Patients with Breast Cancer
Plasma-Exosome	2020	Proteomics	Proteomic Profiling of Plasma and Total Blood Exosomes in Breast Cancer: A Potential Role in Tumor Progression, Diagnosis, and Prognosis
Whole Blood-Exosome	2020	Proteomics	Proteomic Profiling of Plasma and Total Blood Exosomes in Breast Cancer: A Potential Role in Tumor Progression, Diagnosis, and Prognosis
Whole Blood-Exosome	2020	Proteomics	Search for breast cancer proteomic markers in total blood exosomes
Serum-Evs	2019	Proteomics	Proteomic profiling of extracellular vesicles allows for human breast cancer subtyping
Serum-Exosome	2021	Proteomics	Proteomic Landscape of Exosomes Reveals the Functional Contributions of CD151 in Triple-Negative Breast Cancer

MultiOmics studies using liquid biopsy on breast cancer			
**Sample Type**	**Year of Publication**	**Omic**	**Title**
Plasma	2023	Proteomics Genomics	Identification of target proteins for breast cancer genetic risk loci and blood risk biomarkers in a large study by integrating genomic and proteomic data
Plasma	2022	Proteomics Metabolomics	Integrative analysis of plasma metabolomics and proteomics reveals the metabolic landscape of breast cancer
Plasma-EVs	2022	Proteomics Glycomics	Multi-omic analysis of plasma-derived extracellular vesicles in breast cancer
Plasma	2018	Metabolomics Lipidomcs	Multiplatform plasma metabolic and lipid fingerprinting of breast cancer: A pilot control-case study in Colombian Hispanic women
Plasma	2022	Lipidomics, Transcriptomics Genomics	Multi-Omics Approach Points to the Importance of Oxylipins Metabolism in Early-Stage Breast Cancer
Serum	2020	Transcriptomics Metabolomics	Integration of Transcriptome and Metabolome Provides Unique Insights to Pathways Associated With Obese Breast Cancer Patients
Serum	2014	Proteomics Metabolomics	Metabolomic and proteomic analysis of breast cancer patient samples suggests that glutamate and 12-HETE in combination with CA15-3 may be useful biomark[illegible]
Serum	2013	Proteomics Transcriptomics	Coupling proteomics and transcriptomics in the quest of subtype-specific proteins in breast cancer
Serum	2020	Transcriptome Metabolome	Integration of Transcriptome and Metabolome Provides Unique Insights to Pathways Associated With Obese Breast Cancer Patients
Serum	2016	Lipidomics Metabolomics	Two effective models based on comprehensive lipidomics and metabolomics can distinguish BC versus HCs, and TNBC versus non-TNBC
