# Supplementary material for: Biomarker Reproducibility Challenge: A Review of Non-Nucleotide Biomarker Discovery Protocols from Body Fluids in Breast Cancer Diagnosis
Source: Cancers (Basel). 2023 May 16;15(10):2780. doi: 10.3390/cancers15102780 (PMC10216598; doi:10.3390/cancers15102780)
Supplement: Supplementary file 1 [file cancers-15-02780-s001.zip › Supplementary Table S2.pdf]

| Proteomics Study Details | | | | | | | | | | | | | | | | |
|---|---|---|---|---|---|---|---|---|---|---|---|---|---|---|---|---|
| Studeis | | Sample Collection | | | | | Sample inforamtion | | | Method | | Statistical analysis | | | | |
| Referece | Year of Publication | Sample | Sample Amount | Centrifugation | Biosource | Storage | Control (No.) | Breast Cancer (No.) | Cancer Stage | Technique | Validation | Sensitivity | Specificity | Hypothesis test performed | Data availibility | p-value cut off (adjusted) |
| Bartkowiak et al.,2021 | 2021 | Blood in EDTA | 9 ml | 2000 × g for 20 min, 4 °C | Plasma | Short term: −20 °C<br>Long term: −80 °C | • **Training set:** 324 HW<br>• **Validation set: 15** BBD + 103 HW | • **Training set:** 100 BC early detection<br>• **Validation set:** 44 | M0, M1 | ELISA | NA | 80% | 99% | Pearson/Generalized linear model with gamma distribution/Binomial logistic regression/Spearman's rank order correlation | Yes | <0.05 |
| Sun et al., 2016 | 2016 | Blood | 5 ml | 3000 rpm for 5 min + 5 min at 12000 rpm | Serum | −80 °C | 20 | 60 | NA | SELDI-TOF-MS | SDS-PAGE & MALDI-TOF/TOF | NA | NA | Student's t-test | NO | <0.05 |
| Böhm et al., 2011 | 2011 | Blood | NA | 3280 x g for 5 min, 4˚C | Serum | −80 °C | 46 | 45 | NA | SELDI-TOF &<br>MALDI-TOF-TOF | NA | 89% | 67% | t-tests<br>Multivariate discrimination analysis<br>ANOVA<br>ANN<br>ROC | NO | <0.05 |
| Wang et al., 2014 | 2014 | Nipple discharge | 0.5 mL | NA | Nipple discharge | NA | 136 BBD<br>60 HW | 86 | Grade II, III | Electrochemiluminescence | NA | CA15-3: 74.4%<br>CA125: 72.1%<br>CEA: 69.8%<br>TSGF: 69.8 | CA15-3: 82.4%<br>CA125: 83.8%<br>CEA: 86.0%<br>TSGF: 83.1 | Mann-Whitney U-test<br>Kruskal-Wallis<br>Univariate analysis<br>Multivariate analysis | NO | <0.05 |
| Wang et al., 2014 | 2014 | Blood in Heparin | 3ml | NA | Serum | NA | 136 BBD<br>60 HW | 86 | Grade II, III | Electrochemiluminescence | NA | CA15-3: 60.5%<br>CA125: 55.8%<br>CEA: 53.5%<br>TSGF: 62.8 | CA15-3: 91.9%<br>CA125: 90.4%<br>CEA: 89.0%<br>TSGF: 91.2 | Mann-Whitney U-test<br>Kruskal-Wallis<br>Univariate analysis<br>Multivariate analysis | NO | <0.05 |
| Beretov et al., 2015 | 2015 | Urine | 30–50 mL | 2000 x g for 10 min, 4ºC | Supernatant of urine | Short term: −20 °C<br>Long term: −80 °C | 20 HW | 20 | Pre-invasive<br>Early invasive<br>Metastatic | Label-free LC-MS/MS | Western blotting<br>Immunohistochemistry | NA | NA | ANOVA | Yes | <0.05 |
| Gajbhiye et al., 2016 | 2016 | Urine preservative (0.02% w/v sodium azide) | 20 mL | 7000 × *g* for two to three times | Supernatant of urine | NA | **Standardization phase:** 3 HW<br>**Discovery phase:** 24 HW<br>**Validation phase:** 16 HW | **standardization phase:** 3 HE BC<br>**Discovery phase:** 24<br>**Validation phase:** 16 | pT1 / pT2 | Standardization phase: 2-D gel electrophoresis<br>Discovery phase: 2D-DIGE, MALDI-TOF-TOF, SWATH-MS, iTRAQ, LC-QTOF | WB & MRM | NA | NA | Multivariate analysis<br>Chi-square ( 2) | NO | <0.05 |
| Orlanda et al.,2014 | 2014 | Blood | NA | NA | plasma | NA | 121 HW<br>88 BBD | 65 | pT1 / pT2 / pT3 | SELDI-TOF-MS | NA | NA | NA | Wilcoxon–Mann–Whitney<br>Logistic regression<br>Kruskal–Wallis<br>Spearman | NO | <0.05 |
| Arko-Boham et al., 2020 | 2020 | Blood | 5 ml | 2500 rpm for 20 min | Serum | −20 °C | 32 HW | 32 | Grade I-X | ELISA | NA | NA | NA | One-way ANOVA<br>Chi-square | Yes | ≤ 0.05 |
| Tan et al.,2016 | 2016 | Blood | NA | NA | Serum | −80 °C | 120 BBD<br>40 HW | 378 | 0–III | ELISA | NA | 72% | 70% | Mann-Whitney U test<br>Kruskal-Wallis<br>COX regression analysis | NO | < 0.05 |
| Moradpoor et al., 2020 | 2020 | Blood | NA | NA | PBMCs | NA | 3 HW | 21 | NA | LC-MS/MS | Quantitative Real-Time PCR | NA | NA | Student's t-test<br>ANOVA | Yes | < 0.05 |
| Scumaci et al., 2015 | 2015 | Blood in EDTA | 4 ml | 1300 x g for 10 min | Plasma | −80 °C | 4 Healthy family members sharing the same mutation/<br>4 Healthy relatives | 4 Inherited BC | NA | 2D gel analysis<br>LC-MS/MS | Western Blotting | NA | NA | Unpaired t-test | Yes | <0.05 |
| Chen et al.,2017 | 2017 | Blood in EDTA | NA | 4,000 × g for 30 min/ Ultra high speed centrifugation for EV extraction | EV in plasma | −80 °C | 6 HW | 18 | NA | LC-MS/MS | Western Blotting | NA | NA | t-tests | Yes | <0.05 |
| Goufman et al., 2006 | 2006 | Blood | NA | 10,000g for 15 min | Serum thermostable fraction | NA | 10 HW | 10 | I-III | 2D electrophoresis combined with MALDI-TOF-TOF | NA | NA | NA | Kruskal–Wallis<br>Wilcoxon Paired test | NO | <0.05 |
| Hu et al., 2005 | 2005 | Blood | NA | NA | Serum | −80 °C | 51 BBD/ 33 HW | 49 | I–IV | SELDI-TOF-MS | NA | 79.59% | 77.38% | Kruskal–Wallis<br>Fisher exact test<br>Artificial neural networks | NO | <0.05 |

| Studeis | | Sample Collection | | | | | Sample inforamtion | | | Method | | Statistical analysis | | | | |
|---|---|---|---|---|---|---|---|---|---|---|---|---|---|---|---|---|
| Referece | Year of Publication | Sample | Sample Amount | Centrifugation | Biosource | Storage | Control (No.) | Breast Cancer (No.) | Cancer Stage | Technique | Validation | Sensitivity | Specificity | Hypothesis test performed | Data availibility | p-value cut off (adjusted) |
| | | | | | | | | | | | | | | Artificial neural networks | | |
| Zajkowska et al., 2020 | 2020 | Blood in EDTA | NA | 1000×g for 15 min at 2–8°C | Plasma | –85°C | 28 BBD 32 HW | 120 | I-IV | ELISA | NA | 90% | 98.33% | U-Mann Whitney Kruskal-Wallis Multivariate analysis | NO | <0.05 |
| Zajkowska et al., 2020 | 2020 | Blood in EDTA | NA | 1000×g for 15 min at 2–8°C | Plasma | –85°C | 28 BBD 32 HW | 120 | I-IV | Chemiluminescent Microparticle Immuno Assay (CMIA) | NA | 58.33% | 95% | U-Mann Whitney Kruskal-Wallis Multivariate analysis | NO | <0.05 |
| Li et al., 2005 | 2005 | NAF in 10-uL graduated micropipette | NA | 1500 rpm for 10 min | NAF | −80 °C | 5 HW | 5 | I/II | SELDI-TOF-MS | ELISA | NA | NA | Supervised cluster analysis Unsupervised cluster analysis | NO | NA |
| Li et al., 2005 | 2005 | Ductal Lavage Fluid | NA | 1500 rpm for 10 min | Ductal Lavage Fluid | −80 °C | 14 High risk women | 14 Unilateral BC | I/II | SELDI-TOF-MS | ELISA | NA | NA | Supervised cluster analysis Unsupervised cluster analysis | NO | NA |
| TM Pawlik et al., 2005 | 2005 | NAF | NA | 1500 rpm for 10 min | NAF | −80 °C | 5 HW | 23 Unilateral invasive BC | I/II | SELDI-MS | NA | NA | NA | Paired t-tests | NO | <0.05 |
| Noble et al., 2007 | 2007 | NAF | 0 to >100 μl | NA | NAF | −80 °C | 44 HW | 21 Unilateral BC | Grade I-III+ High grade DCIS | SELDI-TOF-MS | NA | NA | NA | Mann–Whitney U-test | NO | <0.05 |
| Sauteret al, 2005 | 2005 | NAF | 1 to 200 μl | NA | NAF | −80 °C | 87 HW | 27 | NA | SELDI-TOF-MS | NA | NA | NA | Two-tailed Fisher's Exact Test Chi square test Kruskal Wallis Test Logistic regression | NO | NA |
| LEBRECHT et al., 2009 | 2009 | Tear in Schirmer strips | NA | NA | Tear | −80 °C | 50 HW | 50 | pT1, pT2, pT3 | SELDI-TOF-MS | NA | ~70% | ~70% | t-test Multivariate statistical analysis Artificial neural networks | NO | <0.05 |
| BÖHM et al., 2012 | 2012 | Tear in Schirmer strips | NA | NA | Tear | −80 °C | 25 HW | 25 | pT1, pT2 | MALDI-TOF-TOF | NA | ~70% | ~70% | In-house-developed algorithm | NO | NA |
| LEBRECHT et al., 2009 | 2009 | Tear in Schirmer strips | NA | NA | Tear | −80 °C | 10 HW | 10 | NA | SELDI-TOF-MS | NA | ~90% | ~90% | t-test Multivariate statistical techniques Artificial neural networks | NO | NA |
| LEBRECHT et al., 2009 | 2009 | Blood | NA | 4000 xg for 5 min | Serum | −80 °C | 10 HW | 10 | NA | SELDI-TOF-MS | NA | ~90% | ~90% | t-test Multivariate statistical techniques Artificial neural networks | NO | NA |
| Oguz Soydinc et al., 2012 | 2012 | Blood | NA | NA | Serum | −20 °C | 60 HW | 180 | NA | ELISA | NA | 86.70% | 70% | Mann Whitney U Spearman's correlation test | NO | <0.05 |
| Oguz Soydinc et al., 2012 | 2012 | 24-hour urine | NA | NA | Urine | −20 °C | 61 HW | 181 | NA | ELISA | NA | 66.70% | 70% | Mann Whitney U Spearman's correlation test | NO | <0.05 |
| Gon Moon et al.,2016 | 2016 | Blood | NA | NA | EV in plasma | NA | **Test:** 20 BBD/ 30 Noncancerousdiseases/ 30 HW **Validation:** 35 BBD/ 50 Noncancerousdiseases/ 40 HW | **Test:** 150 BC/ 40 After surgery **Validation:** 90 BC | 0-IV | ELISA | NA | **Test:** 65.1% **Validation:** 69.2% | **Test:** 83.2% **Validation:** 73.3% | Unpaired t-test Spearman correlation Kruskal-Wallis | NO | <0.05 |
| Garczyk et al., 2015 | 2015 | Blood | 7.5 ml | 1500 g for 10 min at RT | Serum | −80 °C | 40 HW | 40 | pT1-pT4 | ELISA | NA | 64.50% | 89.50% | Mann-Whitney U-test Kruskal-Wallis Fisher's exact test Univariate Kaplan-Meier Multivariate Cox regression | Yes | <0.05 |
| George et al., 2021 | 2021 | NAF | NA | NA | NAF | −80 °C | 4 HW | 9 | pT1-pT3 | LC-MS/MS | NA | NA | NA | Two-sample t-test Pearson Correlation | Yes | <0.05 |
| Corrêa et al., 2017 | 2017 | Blood Sodium EDTA | 20 ml | 1400 × g at for 5 min, 4°C | plasma | NA | Discovery Phase:120 HW Validation Phase: 9 HW | Discovery Phase:107 Validation Phase:37 | Low and High Grade | Label-free Nano-LC/MSMS | Western Blotting | NA | NA | Mann-Whitney | NO | <0.05 |

| Studeis | | Sample Collection | | | | | Sample inforamtion | | | Method | | Statistical analysis | | | | |
|---|---|---|---|---|---|---|---|---|---|---|---|---|---|---|---|---|
| Referece | Year of Publication | Sample | Sample Amount | Centrifugation | Biosource | Storage | Control (No.) | Breast Cancer (No.) | Cancer Stage | Technique | Validation | Sensitivity | Specificity | Hypothesis test performed | Data availibility | p-value cut off (adjusted) |
| | | | | | | | | | | | | | | | | |

NA: Not Available, EDTA: Ethylenediaminetetraacetic Acid, K2 EDTA: Dipotassium Ethylenediaminetetraacetic Acid, h: Hour, min: Minute, RT: Room Temperature, ST: Short-Term, LT: Long-Term, HW: Healthy Woman, BBD: Benign Breast Disease, WB: Western Blotting, ELISA: Enzyme-linked Immunosorbent Assay, ANN: Artificial Neural Network, ROC: Receiver Operating Characteristic.

| Metabolomics Study Details | | | | | | | | | | | | | | | | |
|---|---|---|---|---|---|---|---|---|---|---|---|---|---|---|---|---|
| **Studeis** | | **Sample Collection** | | | | | **Sample inforamtion** | | | **Method** | | **Statistical analysis** | | | | |
| **Referece** | **Year of Publication** | **Sample** | **Sample Amount** | **Centrifugation** | **Biosource** | **Storage** | **Control (No.)** | **Breast Cancer (No.)** | **Cancer Stage** | **Technique** | **Validation** | **Sensitivity** | **Specificity** | **Hypothesis test performed** | **Data availibility** | **p-value cut off (adjusted)** |
| Yuan et al., 2018 | 2018 | Blood in EDTA | NA | **Blood:** 1300 g for 20 min 10°C<br>**Plasma:** 15500 g for 10 min at 10°C | Plasma | −80 °C | **Training:** 100 HW<br>**Validation:** 50 HW | **Training:** 80 BC<br>**Validation:** 109 BC | 0-IV | UHPLC-MS/MS<br>FIA-MS/MS | NA | NA | NA | ASSO regression analysis<br>Univariant logistic regression | Yes | <0.05 |
| Rashed et al., 2020 | 2020 | Blood | 3 mL | 4000 rpm for 5 min | Serum | −20 °C | 55 HW<br>47 BBD | 120 | Early<br>Advanced<br>Metastasis | GC-MS<br>ELISA for CA15-3 | NA | **Combined metabolome with CA15-3:** 88.8% | **Combined metabolome with CA15-3:** 86.8% | Nonparametric analyzes<br>Linear treNA analysis<br>Multiple linear regression<br>stepwise model | NO | <0.05 |
| Hadi et al., 2017 | 2017 | Blood | 4 mL | 4000 rpm for 10 min at 4 °C | Serum | −80 °C | 155 HW | 155 | I-IV | GC-MS | NA | 96% | 100% | Student t-test<br>One way ANOVA<br>Partial Least Square<br>Discriminant Analysis (PLS-DA) | Yes | <0.05 |
| Jove et al., 2017 | 2017 | Blood in EDTA | NA | NA | Plasma | −80 °C | 20 HW | 91 | I-IV | LC-MS | NA | 90%≤ ≤100% | 90%≤ ≤100% | Student T-test<br>Fisher's exact Test<br>Multivariate statistics<br>RaNAom Forest (RF) classifier | Yes | <0.05 |
| Park et al . 2019 | 2019 | Blood in EDTA | NA | 3000 ×g for 10 min at 4°C | Plasma | −80 °C | **Training:** 30 HW<br>**Validation:** 16 HW | **Training:** 40<br>**Validation:** 30 | I–III | LC-MS | NA | NA | NA | Kruskal-Wallis<br>Mann-Whitney U tests | Yes | <0.05 |
| Wei et al., 2021 | 2021 | Blood in EDTA | NA | 1500 xg for 10 min at RT | Plasma | −80 °C | 86 HW | 124 | I–III | LC-QTOF-MS<br>LC-QQQ-MS | NA | 90% | 90% | Student's t test<br>PLS-DA<br>OPLS-DA | Yes | <0.05 |
| Luo et al., 2021 | 2021 | Blood | NA | NA | Serum | −80 °C | 29 BBD | 47 | NA | UPLC-QTOF-MS | NA | 100% | 92.30% | Student's t test<br>Kruskal Wallis H tes<br>Binary logistic regression<br>PLS-DA | Yes | <0.05 |
| Eniu et al., 2018 | 2018 | Blood | 5 mL | 3000 rpm for 10 min at 4°C | Serum | −80 °C | 26 | 30 | I–III | UHPLC-QTOF-(ESI+)-MS | NA | 66%≤ ≤97% | 73%≤ ≤92% | Pearson test<br>PCA<br>PLS-DA | No | <0.05<br>< .01<br>< .001 |
| Kozar et al., 2021 | 2021 | Blood | 5 mL | 2000xG for 10 min at RT | Serum | −80 °C | 21 HW | 39 | I–III | HPLC-TQ/MS | NA | 83% | 81% | PLS-DA<br>Univariate analysis<br>MCCV<br>RaNAom Forest algorithm | Yes | <0.05 |
| Wang et al., 2018 | 2018 | Blood | NA | 3000 g for 10 min at RT | Serum | −80 °C | **Training:** 44 HW/ 38 BBD<br>**Validation:** 30 HW/ 30 BBD | **Training:** 34<br>**Validation:** 30 | NA | UPLC/MS-MS<br>GC-MS/MS | UPLC/MS-MS<br>GC-MS/MS | NA | NA | One-way ANOVA<br>Multivariate statistical analysis<br>PLS-DA | NO | <0.05 |
| Xie et al., 2015 | 2015 | Blood | NA | NA | Plasma/ Serum | −80 °C | **Training:** 35 HW<br>**Validation Set 1:** 41HW<br>**Validation Set 2:** 31HW<br>**Validation Set 3:** 70 | **Training:** 35<br>**Validation Set 1:** 103<br>**Validation Set 2:** 103<br>**Validation Set 3:** 80 | I-IV | LC-TOFMS &<br>GC-TOFMS | NA | 100% | 100% | OPLS-DA<br>Mann-Whitney test<br>logistic regression | NO | <0.05 |
| Jasbi et al., 2018 | 2018 | Blood | NA | NA | Plasma | NA | 99 HW | 102 | I–III | LC-MS/MS | NA | 80% | 75% | Univariate aNA multivariate model<br>Linear model<br>PLS-DA | Yes | <0.05 |
| Suman et al., 2018 | 2018 | Blood in EDTA | NA | 1200×g for 20 min at 4◦C | Plasma | −80 °C | 50 HW | 72 | I-IV | 1H NMR spectroscopy | NA | NA | NA | Multivariate aNA nonparametric statistical analysis | Yes | <0.05 |
| Oktay et al., 2020 | 2020 | Blood | NA | 1200 rcf for 10 min at RT | Plasma | −80 °C | 47 HW | 49 | NA | GC/MS | ELISA | NA | NA | Student's t test<br>RaNAom forest algorithm<br>Pearson's correlation coefficient<br>Logistic regression | Yes | <0.05 |
| Qiu et al., 2013 | 2013 | Blood | NA | NA | Plasma | NA | **Training:** 20 HW<br>**Validation:** 5 HW | **Training:** 30<br>**Validation:** 23 | I-IV | ESI-MS/MS | NA | 98.10% | 96.00% | OPLS-DA<br>Nonparametric univariate method<br>Student's t-test | NO | <0.05 |
| Hu et al., 2016 | 2016 | Urine | NA | NA | Urine | NA | 32 BBD | 33 | 0-IV | Colloid GoldAggregation | NA | NA | NA | Student's t tes | NO | <0.05 |
| Hu et al., 2016 | 2016 | Blood | NA | NA | Plasma | NA | 32 BBD | 33 | 0-IV | LC-MS/M | NA | NA | NA | Student's t tes<br>Wilcoxon's sign rank test | NO | <0.05 |

| Studeis | | Sample Collection | | | | | Sample inforamtion | | | Method | | Statistical analysis | | | | |
|---|---|---|---|---|---|---|---|---|---|---|---|---|---|---|---|---|
| **Referece** | **Year of Publication** | **Sample** | **Sample Amount** | **Centrifugation** | **Biosource** | **Storage** | **Control (No.)** | **Breast Cancer (No.)** | **Cancer Stage** | **Technique** | **Validation** | **Sensitivity** | **Specificity** | **Hypothesis test performed** | **Data availibility** | **p-value cut off (adjusted)** |
| Murata et al., 2019 | 2019 | Saliva in polypropylene tube | 400 μL | NA | Saliva | −80 °C | 42 HW | 101 IC<br>23 DCIS | 0-IV | CE-TOF–MS | LC-QQQ-MS | NA | NA | Mann–Whitney test<br>Kruskal–Wallis test<br>Multiple logistic regression<br>Multiple ADTree models | Yes | <0.05<br>< .01<br>< .001 |
| Zhong et al., 2016 | 2016 | Saliva | 2 mL | 13500 rpm for 20 min at 4°C | Saliva | − 40 °C | 25 HW | 30 | I-IV | UPLC-MS | NA | 48.1%≤ ≤92.6% | 54.2% ≤ ≤100% | Mann-Whitney U test<br>PLS-DA<br>PCA | NO | <0.05 |
| Huang et al., 2012 | 2012 | Urine | NA | NA | Urine | −80 °C | 36 HW | 86 Postmenopausal BC | NA | UFLC-MS/MS | NA | NA | NA | Paired t-test | NO | <0.05 |
| More et al., 2018 | 2018 | Urine<br>(Time not mentioned) | NA | 5000 g for 10 min at 4 °C | Urine | −80 °C | 63 HW | 63 IDC | NA | LC-MRM/MS<br>GC–MS | NA | NA | NA | t-test<br>PCA<br>PLS-DA<br>ROC | NO | <0.05 |
| Lee et al., 2013 | 2013 | Urine<br>(Time not mentioned) | NA | NA | Urine | −20 °C | 11 HW | 6 | NA | GC-MS / LC-MS | NA | NA | NA | PCA<br>PLS-DA<br>t-test<br>ANOVA | NO | <0.05 |
| Slupsky et al., 2010 | 2010 | Urine<br>(Time not mentioned) | NA | NA | Urine | −80 °C | 72 HW | 48 | NA | NMR spectroscopy | NA | NA | NA | PCA, PLS-DA, OPLS-DA<br>Wilcoxon's rank-sum test | NO | <0.05 |
| Cala et al., 2018 | 2018 | First morning Urine | NA | 3000 xg for 10 min at RT | Urine | −80 °C | 29 HW | 31 | I–III | LC-QTOF/MS<br>GC-MS | NA | 93% | 86% | PCA, OPLS-DA<br>Univariate analysis<br>Unpaired t-test<br>Mann −Whitney U test | Yes | <0.05 |
| Silva et al., 2012 | 2012 | Morning Urine | NA | NA | Urine | −80 °C | 21 HW | 26 | NA | HS-SPME & GC–qMS | NA | NA | NA | One way ANOVA<br>LSD test<br>PCA | Yes | <0.05 |
| Woo et al., 2009 | 2009 | Urine<br>(Time not mentioned) | NA | NA | Urine | −20 °C | 12 HW<br>12 Cervical Cancer<br>9 Ovarian Cancer | 10 | NA | GC-MS<br>LC-MS | NA | NA | NA | ANOVA<br>PLS-DA | Yes | <0.05 |
| Zhou et al., 2017 | 2017 | Morning urine | NA | No Centrifugaton | Urine | −80 °C | 11 HW | 11 | I-IV | 1H-NMR | NA | NA | NA | PCA, PLS-DA | NO | <0.05 |
| Do Canto et al., 2016 | 2016 | Ductal lavage fluid | NA | NA | Ductal fluid | −80 °C | 43 Non-affected contralateral breast | 43 Affected breast | 0-III | UPLC-QTOF | NA | 90.70% | 88.40% | Paired t-tests<br>LASSO regression<br>PCA, PLS-DA<br>One way ANOVA | NO | <0.05 |

NA: Not Available, EDTA: Ethylenediaminetetraacetic Acid, K2 EDTA: Dipotassium Ethylenediaminetetraacetic Acid, h: Hour, min: Minute, RT: Room Temperature, ST: Short-Term, LT: Long-Term, HW: Healthy Woman, BBD: Benign Breast Disease, WB: Western Blotting, ELISA: Enzyme-linked Immunosorbent Assay, ANN: Artificial Neural Network, ROC: Receiver Operating C

| Lipidomics Study Details | | | | | | | | | | | | | | | | | |
|---|---|---|---|---|---|---|---|---|---|---|---|---|---|---|---|---|---|
| **Studeis** | | **Sample Collection** | | | | | **Sample inforamtion** | | | **Method** | | | **Statistical analysis** | | | | |
| **Referece** | **Year of Publication** | **Sample** | **Sample Amount** | **Centrifugation** | **Biosource** | **Storage** | **Control (No.)** | **Breast Cancer (No.)** | **Cancer Stage** | **Extraction method** | **Technique** | **Validation** | **Sensitivity** | **Specificity** | **Hypothesis test performed** | **Data availibility** | **p-value cut off (adjusted)** |
| Hammad et al., 2009 | 2009 | Blood | NA | 1200 xg for 12 min at 20˚C | Serum | −80 °C | 25 HW | 50 | IV | Extracted with CHCl3, MeOH aNA H2O. The bottom CHCl3 layer was dried uNAer nitrogen. | Flow injection-ESI-MS3 | NA | NA | NA | Single-factor ANOVA<br>Non-parametric ROC analysis | NO | <0.05 |
| Chen et al., 2016 | 2016 | Blood in EDTA | NA | 2600 xg for 10 minutes at 4°C | Plasma | −80 °C | • **Training Set:** 51 BBC<br>• **Validation Set:** 59 BBC | • **Training Set:** 39<br>• **Validation Set:** 45 | 0-II | Bligh aNA Dyer method with modifications | LC-ESI-MS/MS | Validated | **Training:** 83.3%, **Validation:** 81.0% | **Training:** 92.7%, **Validation:** 94.5% | Binary logistic regression<br>Student's t-test<br>ROC | NO | <0.05 |
| Wolrab et al., 2021 | 2021 | Blood in Lithium-heparin | 9 ml | NA | Plasma | −80 °C | • **Training Set:** 135 HW<br>• **Validation Set:** 57 HW | • **Training Set:** 77<br>• **Validation Set:** 26 | I-IV | Folch method | UHPSFC/MS<br>Shotgun MS | Validated | 91% | 97% | OPLS-DA<br>PCA<br>t-test | Yes | <0.05 |
| Qiu et al., 2013 | 2013 | Blood | NA | NA | Plasma | NA | • **Training Set:** 20 HW<br>• **Validation Set:** 5 HW | • **Training Set:** 30<br>• **Validation Set:** 23 | I-IV | NA | ESI-MS/MS | Validated | 98.10% | 96% | OPLS-DA<br>PCA<br>Student's t-test<br>Binary logical regression | NO | <0.05 |
| Eghlimi et al. 2020 | 2020 | Blood | NA | NA | Plasma | NA | 45 HW | 121 | I-lll | Extracted with MeOH/MTBE | LC-MS/MS | Validated | 96% | 76% | PLS-DA<br>OPLS-DA<br>Two-tailed t-tests<br>ROC | Yes | <0.05 |
| Zhang et al., 2014 | 2014 | Blood | NA | NA | Serum | NA | • **Training Set:** 70 HW<br>• **Validation Set:** 132 HW | • **Training Set:** 68<br>• **Validation Set:** 72 | I-IV | Extracted with MeOH/CAN | FTICR<br>MS | Validated | 83.30% | 87.10% | Mann–Whitney U test<br>ROC | Yes | <0.05 |
| Cui et al., 2016 | 2016 | Blood | NA | NA | Serum | NA | 20 HW | 20 | I-III | Extracted with MeOH | LC-QTOF-MS/MS | Validated | NA | NA | Wilcoxon<br>Mann–Whitney<br>PLS-DA<br>OPLS-DA | NO | <0.05 |
| Yang et al., 2015 | 2015 | Blood | NA | NA | Plasma | NA | 9 HW<br>6 BBC | 5 | NA | Bligh aNA Dyer with modifications | NP/RP 2D LC-MS | NA | NA | NA | Mann–Whitney U test<br>PCA | NO | <0.05 |
| Jiang et al., 2017 | 2017 | Blood in heparin | 10 ml | 1500 xg for 15 min | Plasma | −80 °C | 41 HW | 37 | I–II | Extracted with Isopropanol | UPLC-QTOF/MS | NA | NA | NA | One-way ANOVA<br>t-tests<br>OPLS-DA<br>Univariate statistical analysis | NO | <0.05 |
| Min et al., 2010 | 2010 | Urine | NA | NA | Lyophilized Urine | NA | 5 HW | 5 | NA | Folch method | Nanoflow LC-ESI-MS/MS | NA | NA | NA | NA | NO | NA |
| Kim et al., 2009 | 2009 | Urine | NA | NA | Lyophilized Urine | NA | 5 HW | 5 | NA | Folch method | Nanoflow LC-ESI-MS/MS | NA | NA | NA | NA | NO | NA |
| Cala et al., 2018 | 2018 | First-pass urine | NA | 3000 x g for 10 min at room temperature | Supernatant of urine | −80 °C | 29 HW | 31 | I-III | Extracted with MeOH/MTBE/Deionized water | LC–MS | NA | 93% | 86% | OPLS-DA<br>Unpaired t-test<br>Mann −Whitney U test<br>ROC | Yes | <0.05 |
| | | [illegible] | | | | | | | | | | | | | Mann–Whitney U-test | | |

| Studeis | | Sample Collection | | | | | Sample inforamtion | | | Method | | | Statistical analysis | | | | |
|---|---|---|---|---|---|---|---|---|---|---|---|---|---|---|---|---|---|
| Referece | Year of Publication | Sample | Sample Amount | Centrifugation | Biosource | Storage | Control (No.) | Breast Cancer (No.) | Cancer Stage | Extraction method | Technique | Validation | Sensitivity | Specificity | Hypothesis test performed | Data availibility | p-value cut off (adjusted) |
| Bel'skaya et al., 2021 | 2021 | Saliva in polypropylene tubes | 1 ml | 10,000× g for 10 min | Saliva | Without freezing and storage | 100 HW | 30 | T2N0-1 T3N0-1 | Folch method | IR spectroscopy | NA | NA | NA | Kruskal–Wallis test Discriminant analysis | NO | NA |

NA: Not Available, EDTA: Ethylenediaminetetraacetic Acid, K2 EDTA: Dipotassium Ethylenediaminetetraacetic Acid, h: Hour, min: Minute, RT: Room Temperature, ST: Short-Term, LT: Long-Term, HW: Healthy Woman, BBD: Benign Breast Disease, WB: Western Blotting, ELISA: Enzyme-linked Immunosorbent Assay, ANN: Artificial Neural Network, [illegible]
